# Caesarean section in pregnancies conceived by assisted reproductive technology: a systematic review and meta-analysis

**DOI:** 10.1186/s12884-021-03711-x

**Published:** 2021-03-22

**Authors:** Nakeisha A. Lodge-Tulloch, Flavia T. S. Elias, Jessica Pudwell, Laura Gaudet, Mark Walker, Graeme N. Smith, Maria P. Velez

**Affiliations:** 1 Department of Obstetrics and Gynecology, Kingston General Hospital, Queen’s University, Victory 4, 76 Stuart St, Kingston, Ontario K7L 2V7 Canada; 2 Health Technology Assessment Program, Oswaldo Cruz Foundation, Brasilia, Brazil; 3 Department of Obstetrics, Gynecology & Newborn Care, University of Ottawa, 451 Smyth Road, Ottawa, Ontario K1H 8M5 Canada; 4 Department of Public Health Sciences, Queen’s University, 62 Fifth Field Company Lane, Kingston, Ontario K7L 3N6 Canada

**Keywords:** Elective caesarean section, Emergent caesarean section, Frozen embryo transfer, Fresh embryo transfer, In-vitro fertilization (IVF), Intracytoplasmic sperm injection (ICSI), Maternal outcomes

## Abstract

**Background:**

Caesarean section rates are higher among pregnancies conceived by assisted reproductive technology (ART) compared to spontaneous conceptions (SC), implying an increase in neonatal and maternal morbidity. We aimed to compare caesarean section rates in ART pregnancies versus SC, overall, by indication (elective versus emergent), and by type of ART treatment (in-vitro fertilization (IVF), intracytoplasmic sperm injection (ICSI), fresh embryo transfer, frozen embryo transfer) in a systematic review and meta-analysis.

**Methods:**

We searched Medline, EMBASE and CINAHL databases using the OVID Platform from 1993 to 2019, and the search was completed in January 2020. The eligibility criteria were cohort studies with singleton conceptions after in-vitro fertilization and/or intracytoplasmic sperm injection using autologous oocytes versus spontaneous conceptions. The study quality was assessed using the Newcastle Ottawa Scale and GRADE approach. Meta-analyses were performed using odds ratios (OR) with a 95% confidence interval (CI) using random effect models in RevMan 5.3, and I-squared (I^2^) test > 75% was considered as high heterogeneity.

**Results:**

One thousand seven hundred fifty studies were identified from the search of which 34 met the inclusion criteria. Compared to spontaneous conceptions, IVF/ICSI pregnancies were associated with a 1.90-fold increase of odds of caesarean section (95% CI 1.76, 2.06). When stratified by indication, IVF/ICSI pregnancies were associated with a 1.91-fold increase of odds of elective caesarean section (95% CI 1.37, 2.67) and 1.38-fold increase of odds of emergent caesarean section (95% CI 1.09, 1.75). The heterogeneity of the studies was high and the GRADE assessment moderate to low, which can be explained by the observational design of the included studies.

**Conclusions:**

The odds of delivering by caesarean section are greater for ART singleton pregnancies compared to spontaneous conceptions. Preconception and pregnancy care plans should focus on minimizing the risks that may lead to emergency caesarean sections and finding strategies to understand and decrease the rate of elective caesarean sections.

**Supplementary Information:**

The online version contains supplementary material available at 10.1186/s12884-021-03711-x.

## Background

Infertility, defined as the inability to conceive after 12 or more months of regular unprotected intercourse, affects 12–15% of couples [1, 2]. Between 1 and 5% of children in industrialized countries are born following assisted reproductive technologies (ART) [3]. ART has been associated with higher caesarean section rates compared to women who conceive spontaneously [4].

The overall rate of caesarean sections continues to increase at a rapid rate. The ideal caesarean section rate is 10–15% according to the World Health Organization (WHO) [5], which states that population level rates higher than 10% are not associated with reductions in maternal and neonatal mortality [5]. Globally, the rate of caesarean section has increased from 12.1% in 2000 to 21.1% in 2015 [6].

Previous studies have compared caesarean sections between fresh and frozen embryo transfer in ART pregnancies [7], in oocyte donation pregnancies [8], and in multiple pregnancies conceived by IVF [9, 10]. Two systematic reviews and meta-analyses published in 2004 estimated an increased risk of caesarean delivery among the IVF/ICSI population [11, 12], followed by a third meta-analysis published in 2012 which confirmed those findings [4]. However, the identification of associated treatment factors has not been addressed in previous meta-analyses. This can help to establish care plans for women undergoing ART to improve pregnancy deliveries and to reduce possible harm in unnecessary caesarean sections in these pregnancies.

The objective of the present study is to conduct a systematic review and meta-analysis to assess the risk of caesarean section in IVF/ICSI singleton pregnancies compared to spontaneous conceptions, overall and by indication (elective versus emergent), and by type of ART treatment (IVF, ICSI, fresh embryo transfer, and frozen embryo transfer).

## Methods

### Search strategy and information sources

We conducted a literature search from 1993 to 2019 on MEDLINE, EMBASE and the cumulative index to nursing and allied health literature (CINAHL) database using the OVID Platform. The search was completed in January 2020. MeSH terms and the indexing of terms were used. The keywords used in database searches were; in vitro fertilization/or intracytoplasmic sperm injection/, fertilization in vitro, in vitro fertilization*.mp., reproductive techniques assisted, caesarean section/ or repeat caesarean section/, cesarean section*mp., ceasarean section*.mp., caesarean section*.mp., c-section*.mp., caesarean delivery, caesarean section, elective. Keywords with the notation “*mp” indicate the plural form of that term was searched, and the term was also searched as a keyword (See supplementary materials, Additional file 1). Additionally, search criteria included studies after 1990 limited to only English and French literature and grey literature. References of past systematic reviews were also searched for relevant articles to include in the review. The PRISMA (Preferred Reporting Items for Systematic Reviews and Meta-analyses) Statement [13] was followed in preparation of this manuscript. PROSPERO register (CRD42020165075).

### Study selection and eligibility criteria

Two team members independently performed the title and abstract screening and conducted full text screening (NAL, FTSE). Conflicts were resolved by consensus or by a third team member (MPV). Criteria to identify eligible publications for the current review were established using the PICOS (Population-Intervention- Comparators-Outcomes-Study design) framework. The inclusion criteria were singleton pregnancies conceived using ART (IVF and/or ICSI) with autologous oocytes compared to spontaneously conceived singleton pregnancies. The exclusion criteria were pregnancies conceived using intrauterine insemination (IUI), exclusive ovulation induction, or IVF/ICSI using donor gametes (oocyte, embryo or sperm), gestational surrogacy and twins or higher order multiples pregnancies.

### Exposure and outcome measures

The main exposure was IVF and/or ICSI combined. Additional analyses were conducted by type of fertilization (IVF or ICSI), and type of embryo transfer (fresh or frozen). The outcomes of interest were caesarean section, overall and by indication (e.g. elective and emergent caesarean section). We used the Lucas et al. classification of urgency of caesarean section [14], grouped as emergent (grade I: emergent and grade II: urgent) and elective (grade III: scheduled and grade IV: elective). Most literature classifies caesarean section as elective or emergent, where an elective caesarean section is one performed for nonclinical reasons and an emergent caesarean section is one performed due to an immediate threat to the life of the woman or fetus [14].

### Assessment of heterogeneity

The similarity between the included studies (mainly regarding study design and clinical characteristics) was assessed to ensure pooling was appropriate. The I^2^ statistic was used to analyze heterogeneity. High heterogeneity is indicated by a percentage greater than 75%.

### Risk of bias and quality assessment

Risk of bias and quality assessment of included studies was performed independently by two authors (NAL, FTSE). Conflicts were resolved by consensus or by a third team member (MPV). Study quality was assessed by two reviewers using the Newcastle-Ottawa Scale (NAL, FTSE). This system involves eight scored items, each included study was evaluated in these categories and received a total score ranging from 0 to 9 points. A score of 8 or 9 indicates a high-quality study, a score of 6 to 7 indicates a moderate quality study, and < 5 low quality study [15]. Publication bias was assessed by Funnel Plot graphics using RevMan 5.3 software if the pooled analysis included more than 10 studies (Additional file 2) [16]. In addition, a senior investigator (FTSE) applied the GRADE (Grading of Recommendations Assessment, Development, and Evaluation) approach to rate the quality of the evidence using GRADE Profiler (GRADEpro), version 3.6 [17].

### Statistical analysis

Data extracted from included studies was composed into 2 × 2 tables to conduct a meta-analysis using RevMan 5.3 software. Studies with similar outcomes were pooled together and the tables were used to calculate crude odds ratios. For the outcome of caesarean section, measures of association were reported as odds ratios with a 95% confidence interval. Data was analyzed using the random effect model which assumes heterogeneity and the significance of the pool odds ratio was analyzed using the Mantel-Haenszel statistical method. When conducting the meta-analysis, the number of individuals undergoing caesarean section for five studies [18–22] needed to be estimated based on percentages provided as no explicit number was stated in the study.

This systematic review and meta-analysis did not involve consumer and community participation. The study was approved by the Queen’s University Health Sciences & Affiliated Teaching Hospitals Research Ethics Board on October 29, 2019 (Reference number OBGY-357-19). Additionally, informed consent was not applicable in this study as there were no human participants involved.

## Results

### Search results

**Fig. 1:**
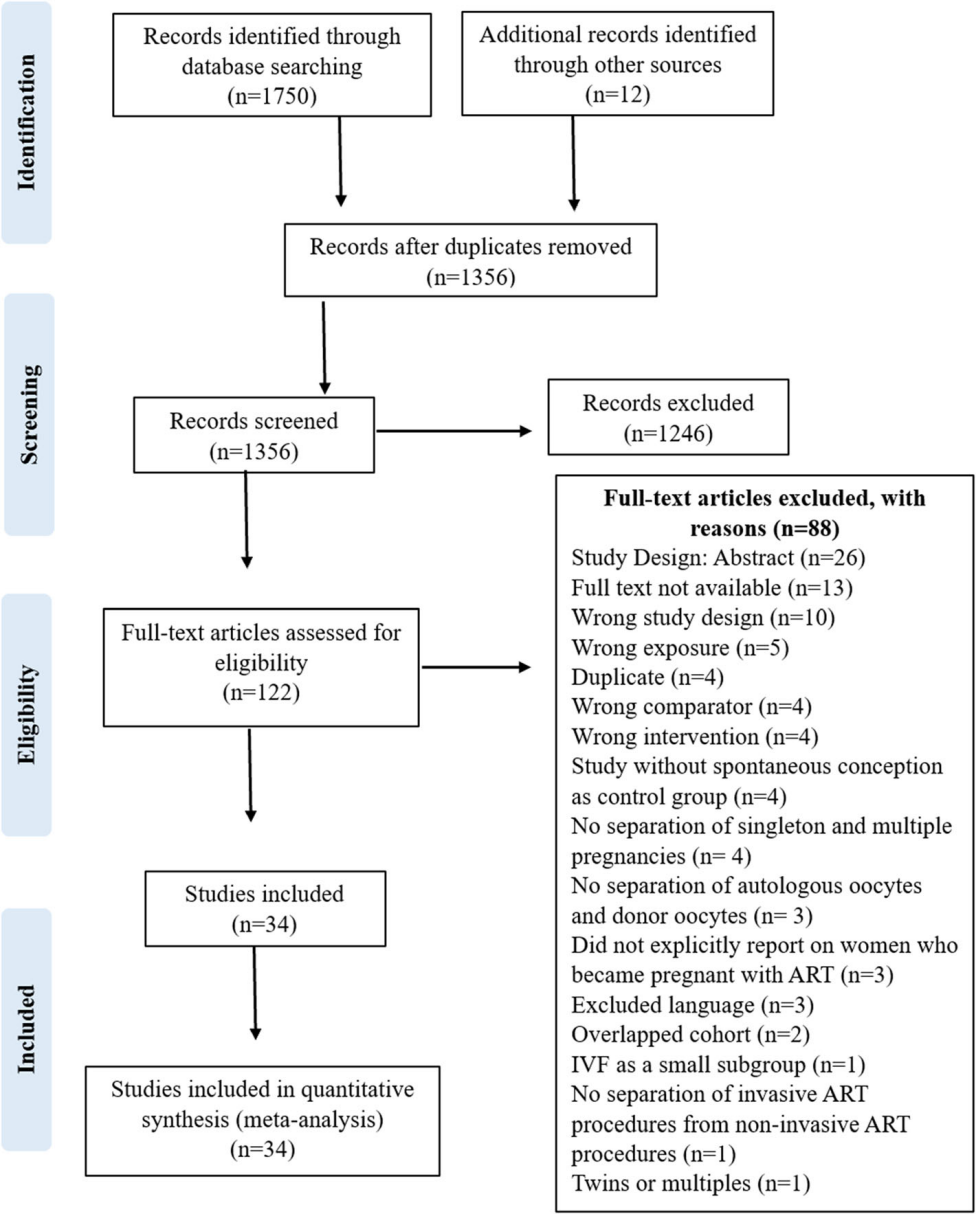
PRISMA Flowchart. Flowchart showing identification and selection of studies included in the systematic review and meta-analysis

There were a total of 1750 studies resulting from the search of MEDLINE, EMBASE and CINAHL databases. An additional 12 studies were identified through manual examination of the references from the initial search. Figure 1 displays the process of screening and selecting the studies for the review and meta-analysis. During full text screening, three studies were identified as having used the same cohort study and as such the most recent study was included [23] and the other two studies removed [24, 25]. A total of 34 studies were included in the review and meta-analysis, of which 17 were matched cohort studies [18, 20, 23, 26–39] and 17 unmatched cohort studies [19, 21, 22, 40–53]. Excluded manuscripts are listed in Additional file 3.

### Study characteristics

The characteristics of each study selected for this review are presented in Table 1. Studies were conducted in Europe (*n* = 23), Canada (*n* = 3), The United States (n = 2) and Asia (*n* = 6). Twenty-nine studies were retrospective cohort studies [18, 20, 21, 23, 26–28, 30–43, 46–53], while five were prospective cohort studies [19, 22, 29, 44, 45]. Twenty-two of the selected studies were hospital based cohort studies [19–22, 26–29, 32–34, 36–41, 44, 46, 51–53], while twelve of the selected studies were population based cohort studies [18, 23, 30, 31, 35, 42, 43, 45, 47–50]. Fourteen studies provided data on exclusively IVF procedures [18, 20–22, 28–30, 32, 35, 39, 43, 44, 46, 47], and six studies on ICSI procedures [18, 19, 28, 35, 44, 47]. Seven studies reported data on fresh embryo transfer [23, 26, 29, 35, 40, 43, 50], and six reported frozen embryo transfer [23, 26, 35, 40, 43, 50]. Additionally, ten studies reported on the type of caesarean section performed, either elective or emergent [20, 30, 34, 36, 37, 39, 41, 47, 48, 51].

**Table 1:** Characteristics of Included Studies

Study ID and Location	Study Design	Number of Participants, n (singletons)	Maternal Age, mean ± SD number or %	Exposure	NOS Score
Anzola et al. (2019) [26] France	Retrospective hospital- based/ Matched	Fresh ET = 5883 Frozen ET= 366 SC = 6981	Fresh ET 32.8 ± 4.4 Frozen ET 33.1 ± 4.3 SC not available	IVF and IVF + ICSI, Fresh ET or Frozen ET	8
Apantaku et al. (2008) [27] England	Retrospective hospital- based/ Matched	ART = 88 SC = 88	ART 33.5 + 4.0 SC-33.2 + 4.1	IFV/ICSI	8
Beyer et al. (2016) [40] Germany	Retrospective hospital- based	ART = 467 SC = 6417	ART 33.7 + 4.0 SC 28	ART (Fresh ET, Slow-rate Freezing, Vitrification)	7
Buckett et al. (2007) [28] Canada	Retrospective hospital- based/ Matched	IVF = 133 ICSI = 104 SC = 338	IVF 35 ICSI- 34 SC 34	Fresh ET after IVF Fresh ET after ICSI	9
Carbillon et al. (2017) [41] France	Retrospective hospital- based	IVF/ICSI = 119 SC = 7993	Mild ovarian stimulation with ART- 30.2 + 5.9 Multi-follicular stimulation and ART- 29.9 + 6.0 SC - 29.7 + 5.8	IFV/ICSI (Excluded oocyte donation, previous diabetes)	7
Dayan et al. (2018) [42] Canada	Retrospective population-based	IVF = 1596 SC = 112,813	IVF 35.7 ± 4.6 SC 30.3 ± 5.2	IVF including (ICSI both fresh and frozen embryo transfer)	9
D’Souza et al. (1997) [29] England	Prospective hospital-based/Matched	IVF = 150 SC = 150	Not available	Fresh ET after IVF	9
Ensing et al. (2015) [30] Netherlands	Retrospective population-based/ Matched/ adjusted	IVF = 16,177 SC = 1,905,011	IFV 32.7 + 4.6 SC 32.7 + 4.5	IVF as a subgroup	9
Ernstad et al. (2016) [43] Sweden	Retrospective population-based	Fresh ET = 22,771 Frozen ET = 7795 SC = 1,196,394	> 35 years All blastocyst (47.1%) All cleavage (45.7%) SC (20.4%)	Fresh ET after IVF Frozen ET after IVF (excluded oocyte donation)	7
Farhi et al. (2013) [44] Israel	Prospective hospital-based	IVF = 202 ICSI = 307 SC = 587	ART - 33.1 ± 4.9 SC - 30.1 ± 4.9	ART (IVF and ICSI)	6
Fedder et al. (2013) [18] Denmark	Retrospective population-based/ Matched/ adjusted	ICSI = 6156 IVF = 11,060 SC = 33,852	ICSI (Group A = 32.78 + 4.27, Group B = 33.16 + 4.05) IVF (Group C): 34.01 + 4.04 SC (Group D): 30.23 + 4.86	IVF and ICSI	9
Gambadauro et al. (2017) [45] Sweden	Prospective population-based	IVF = 167 SC = 3116	> 35 years IVF 46.1% SC 22.6%	IVF with or without ICSI	7
Gillet et al. (2011) [31] Belgium	Retrospective population-based/Matched	IVF/ICSI = 1866 SC = 15,228	IVF/ICSI − 37.8 ± 2.4 SC 37.3 ± 2.0	IFV/ICSI	9
Harlev et al. (2018) [46] Israel	Retrospective hospital- based	IVF = 229 SC = 7929	IVF 41 + 1.35 SC 41 + 1.20	IVF and Ovulation induction	9
Katalinic et al. (2004) [19] Germany	Prospective hospital-based	ICSI = 2055 SC = 7861	ICSI 32.9 + 3.9 SC 27.0 + 4.7	Fresh embryo transfer (ET) after ICSI	7
Koudstaal et al. (2000) [20] Netherlands	Retrospective hospital- based/ Matched	IVF = 307 SC = 307	IVF 32.8 (+ 4.3) SC 32.7 + (4.4)	Fresh ET after IVF (excluded frozen and embryo reduction)	9
Liu et al. (2015) [53] China	Retrospective hospital- based	IVF = 380 SC = 405	IVF/ICSI 31.59 ± 3.48 years SC 31.31 ± 3.45 years	IVF/ICSI	4
Malchau et al. (2014) [47] Denmark	Retrospective population-based	IVF = 4135 ICSI = 3635 SC = 229,749	IVF 34.2 (+ 4.4) ICSI 33.4 (+ 4.3) SC 30.7 (+ 4.9)	IUI IVF and ICSI	9
Ochsenkuhn et al. (2003) [32] Germany	Retrospective hospital- based/ Matched/ adjusted	IVF = 163 SC = 322	IVF 32.6 SC 32.2	IVF GIFT	9
Olivennes et al. (1993) [21] France	Retrospective hospital- based	IVF = 162 SC = 5096	IVF 33.6 ± 3.9 SC 29.9 ± 4.7	IVF	7
Olson et al. (2005) [33] United States	Retrospective hospital- based/ Matched	IVF/ICSI = 645 SC = 4590	IVF 33.9 + 4.6 SC 33.3 + 4.3	IVF/ICSI	8
Perri et al. (2001) [34] Israel	Retrospective hospital- based/ Matched	ART = 95 SC = 190	ART 32.15 + 4.5 SC 32.13 + 4.5	IFV/ICSI Transferring both IVF- and ICSI-derived embryos	9
Pinborg et al. (2010) [35] Denmark	Retrospective population-based/ Matched/ adjusted	Frozen = 957 Fresh =10,329 Non-ART = 4800	Frozen 34.0 (3.8) Fresh 33.7 (4.0) Non-ART 30.1 (4.8)	Frozen ET after IVF/ICSI Fresh ET after IVF/ICSI	8
Poikkeus et al. (2007) [36] Finland	Retrospective hospital- based/ Matched/ adjusted	IVF/ICSI = 499 (SET = 269; DET = 230) SC = 15,037	Single ET 32.6 + 3.9 Double ET 34.2 + 3.8 SC 30.3 + 5.3	IFV/ICSI (Single ET or Double ET)	8
Rahu et al. (2019) [48] Estonia	Retrospective population-based	IVF/ICSI = 1778 SC = 33,555	IVF/ICSI 32.5 ± 3.8 SC 28.6 ± 3.3	IVF/ICSI, autologous	9
Romundstad et al. (2008) [49] Norway	Retrospective population-based	ART = 8229 SC = 1,200,922	All ranges, and ≥35 years ART 35% SC 12%	IFV/ICSI	9
Sazonova et al. (2012) [50] Sweden	Retrospective population-based	Frozen = 2348 Fresh = 8944 SC = 571,914	n 30–39 ART = 8754 SC = 297,818 *n* ≥ 40 ART = 836 SC = 18,096	Frozen ET after IVF/ICSI Fresh ET after IVF/ICSI (excluded oocyte donation)	9
Shevell et al. (2005) [22] United States	Prospective hospital-based	ART = 554 SC = 34,286	IVF 34.5 (+ 5.2) SC 29.9 (+ 5.7)	IVF ICSI GIFT/ZIFT	8
Stojnic et al. (2013) [37] Serbia	Retrospective hospital- based/ Matched / adjusted	IVF = 351 ICSI = 283 SC = 634	IVF/ICSI 36 ± 4.2 SC 35 ± 4.1	IVF/ICSI (excluded oocyte donation, frozen and vanishing twins)	9
Sun et al. (2014) [38] Canada	Retrospective hospital- based/ Matched/adjusted	ART = 1327 SC = 5222	All ranges, and ≥ 35 years: ART 51% SC 50%	IFV/ICSI	9
Suzuki et al. (2007) [51] Japan	Retrospective hospital- based	IVF-ET = 89 SC = 849	All ≥35 years	IVF/ICSI	8
Tomic et al. (2011) [39] Croatia	Retrospective hospital- based/ Matched	IVF = 283 SC = 283	IVF = 37.8 ± 3.9 SC = 37.4 ± 3.8	IVF in advanced age (excluded oocyte donation, cryopreservation, vanishing twins)	9
Toshimitsu et al. (2014) [52] Tokyo	Retrospective hospital- based	IVF/ICSI = 116 SC = 662	IVF/ICSI41.5 ± 1.5 SC 41.2 ± 1.4	IVF/ICSI, autologous	7
Wennerholm et al. (2013) [23] Denmark, Norway and Sweden	Retrospective population-based/Matched/ adjusted	Frozen ET = 6647 Fresh ET = 42,242 SC = 288,542	Frozen: 33.7 + 3.9 Fresh: 33.3 + 4.0 SC:28.5 + 5.0	Frozen ET after IVF/ICSI	9

*NOS* Newcastle Ottawa Scale, *SC* Spontaneous Conception, *IUI* Intrauterine Insemination, *ART* Assisted Reproductive Technology, *IVF* In Vitro Fertilization, *ICSI* Intracytoplasmic Sperm Injection, *ET* Embryo Transfer, *SET* Single Embryo Transfer, *DET* Double Embryo Transfer, *GIFT* Gamete Intrafallopian Transfer

#### IVF/ICSI versus spontaneous conception

**Fig. 2:**
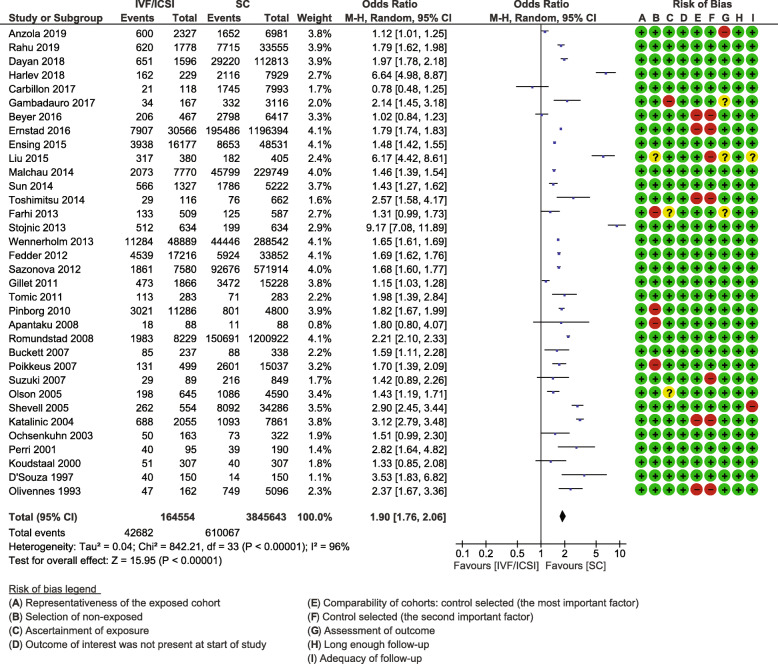
Caesarean Section Meta-analysis. Forest plot displaying the results of a meta-analysis comparing caesarean sections after in vitro fertilization (IVF) / intracytoplasmic sperm injection (ICSI) versus spontaneous conception

Thirty-four studies met the inclusion criteria [18–23, 26–53], resulting in 164,603 pregnancies following IVF/ICSI compared to 3,845,643 spontaneous conceptions. The pooled OR was 1.90 (95% CI 1.76, 2.06) with high heterogeneity between the studies I^2^ = 96% (Fig. 2). Seventeen studies were matched or adjusted cohorts [18, 20, 23, 26–39], and seventeen unmatched cohorts [19, 21, 22, 40–53]. The quality of individual studies according to the NOS was moderate to high with NOS scores ranging from 4 to 9, of which 25 studies had scores of 8 or 9 (Table 1). Publication bias was low as demonstrated by a funnel plot with symmetric distribution (Additional file 2, Fig. 1). The GRADE quality assessment was moderate (Additional file 4).

##### Elective caesarean section

**Fig. 3:**
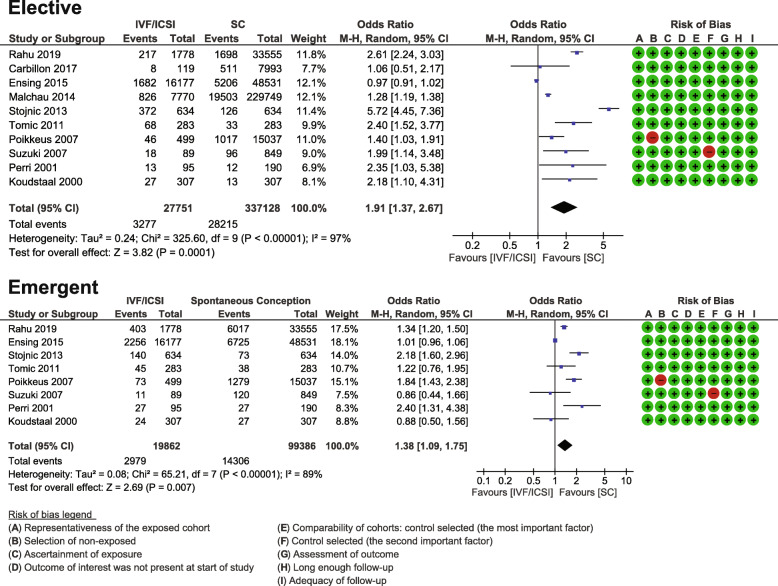
Elective Caesarean Section and Emergent Caesarean Section Meta-analyses. Forest plots displaying the results of meta-analyses comparing elective caesarean sections after in vitro fertilization (IVF) / intracytoplasmic sperm injection (ICSI) treatment versus spontaneous conception and comparing emergent caesarean sections after in vitro fertilization (IVF) / intracytoplasmic sperm injection (ICSI) treatment versus spontaneous conception

Ten studies met the inclusion criteria and reported data on elective caesarean sections (*n* = 27,799 pregnancies following IVF/ICSI compared to 337,128 spontaneous conceptions) [20, 30, 34, 36, 37, 39, 41, 47, 48, 51]. The pooled OR was 1.91 (95% CI 1.37, 2.67) with high heterogeneity between the studies I^2^ = 97% (Fig. 3). Six studies were matched cohorts [20, 30, 34, 36, 37, 39], and four unmatched cohorts [41, 47, 48, 51]. The quality of studies was moderate to high with NOS scores ranging from 7 to 9 (Table 1). The GRADE quality assessment was moderate (Additional file 4).

##### Emergent caesarean section

Eight studies also met the inclusion criteria and reported data on emergent caesarean sections (*n* = 19,862 pregnancies following IVF/ICSI compared to 99,386 spontaneous conceptions) [20, 30, 34, 36, 37, 39, 48, 51]. The pooled OR was 1.38 (95% CI 1.09,1.75) with high heterogeneity between the studies I^2^ = 89% (Fig. 3). Six studies were matched cohorts [20, 30, 34, 36, 37, 39], and two unmatched cohorts [48, 51]. The quality of studies was high with NOS scores ranging from 8 to 9 (Table 1). The GRADE quality assessment was moderate (Additional file 4).

#### In vitro fertilization (IVF) versus spontaneous conception

**Fig. 4:**
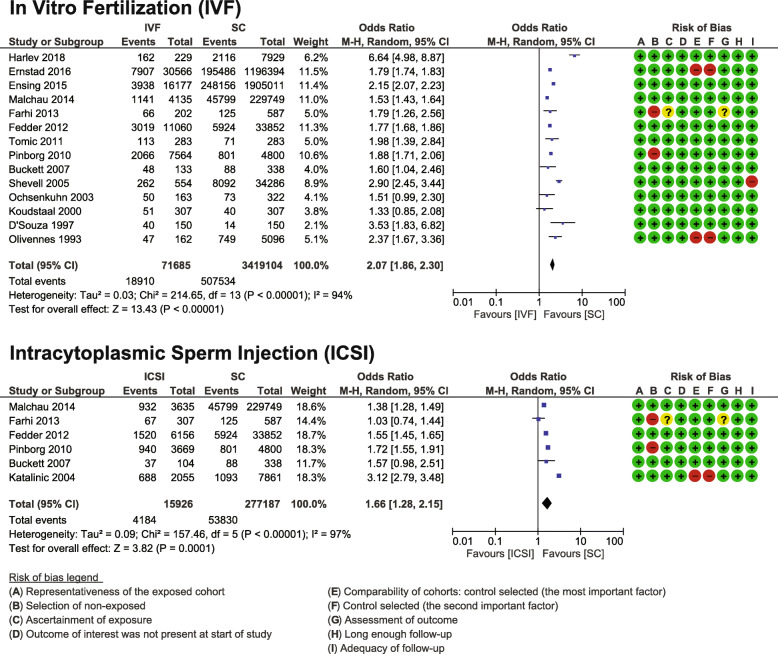
Fertilization Mode (**a** IVF, **b** ICSI) Meta-analysis. Forest plot displaying the meta-analyses comparing caesarean sections after exclusively in vitro fertilization procedures versus spontaneous conception and comparing exclusively intracytoplasmic sperm injection procedures versus spontaneous conception

Fourteen studies met the inclusion criteria (*n* = 71,685 IVF pregnancies vs. 3,419,104 spontaneous conceptions) [18, 20–22, 28–30, 32, 35, 39, 43, 44, 46, 47]. The pooled OR was 2.07 (95% CI 1.86, 2.30) with high heterogeneity between the studies I^2^ = 94% (Fig. 4). Eight studies were matched cohorts [18, 20, 28–30, 32, 35, 39], and six unmatched cohorts [21, 22, 43, 44, 46, 47]. The quality of studies according to the NOS was moderate to high with NOS scores ranging from 6 to 9 (Table 1). The GRADE quality assessment was moderate (Additional file 4).

#### Intracytoplasmic sperm injection (ICSI) versus spontaneous conception

Six studies met the inclusion criteria (*n* = 15,926 ICSI pregnancies vs. 277,187 spontaneous conceptions) [18, 19, 28, 35, 44, 47]. The pooled OR was 1.66 (95% CI 1.28, 2.15) with high heterogeneity between the studies I^2^ = 97% (Fig. 4). Three studies were matched cohorts [18, 28, 35], and three unmatched cohorts [19, 44, 47]. The quality of studies according to the NOS was moderate to high with NOS scores ranging from 6 to 9 (Table 1). The GRADE quality assessment was moderate (Additional file 4).

#### Fresh embryo transfer versus spontaneous conception

**Fig. 5:**
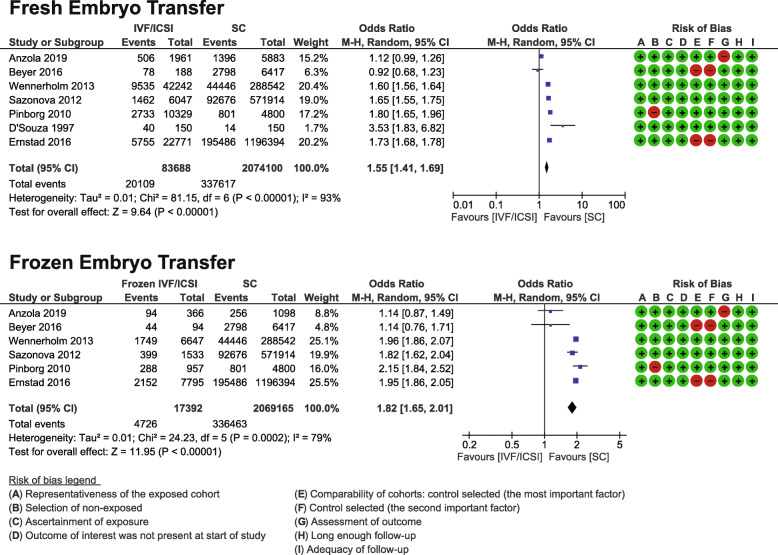
Fresh and Frozen in vitro fertilization (IVF) / intracytoplasmic sperm injection (ICSI) Meta-analysis. Forest plot displaying the meta-analyses comparing caesarean sections after frozen in vitro fertilization (IVF) / intracytoplasmic sperm injection (ICSI) treatment versus spontaneous conception and comparing fresh in vitro fertilization (IVF) / intracytoplasmic sperm injection (ICSI) treatment versus spontaneous conception

Seven studies met the inclusion criteria (*n* = 83,688 following fresh embryo transfer compared to 2,074,100 spontaneous conceptions) [23, 26, 29, 35, 40, 43, 50]. The pooled OR was 1.55 (95% CI 1.41, 1.69) with high heterogeneity between the studies I^2^ = 93% (Fig. 5). Three studies were matched cohorts [23, 26, 35], and two unmatched cohorts [40, 50]. The quality of studies was moderate to high with NOS scores ranging from 7 to 9 (Table 1). The GRADE quality assessment was low (Additional file 4).

#### Frozen embryo transfer versus spontaneous conception

Six studies met the inclusion criteria (*n* = 17,392 pregnancies following frozen embryo transfer compared to 2,069,165 spontaneous conceptions) [23, 26, 35, 40, 43, 50]. The pooled OR was 1.82 (95% CI 1.65, 2.01) with high heterogeneity between the studies I^2^ = 79% (Fig. 5). Three studies were matched cohorts [23, 26, 35], and two unmatched cohorts [40, 50]. The quality of studies was moderate to high with NOS scores ranging from 7 to 9 (Table 1). The GRADE quality assessment was low (Additional file 4).

## Discussion

### Main findings

Our study indicates that IVF/ICSI pregnancies are associated with higher odds of caesarean section compared to spontaneous conceptions. The odds were also greater for elective caesarean sections compared to spontaneous conceptions than for emergent caesarean sections. This trend was also apparent, in IVF or ICSI, and fresh or frozen embryo transfer, compared to spontaneous conception. Our study presents updated rates of caesarean section between ART and spontaneous pregnancies, with 16 studies conducted after 2012. In addition, we considered type of treatment (IVF, ICSI, fresh, and frozen embryo transfer) as independent factors. A strength of the study is the type of included studies. While the quality scores ranged from low to high with scores from 4 to 9, 25 studies (75%) were considered high quality studies. Furthermore, majority of the included studies, with the exception of two studies, considered potential confounders in the analysis. According to the GRADE approach, the quality of the caesarean section effect estimate, overall, by indication (emergent, elective), IVF, or ICSI was moderate, while it was low for Fresh or Frozen embryo transfer. The high heterogeneity (I^2^ > 75%) and low GRADE scores in some of the subgroup analyses can be explained by variations in the definition of the outcomes and/or indication of emergent or elective caesarean section, and inclusion and exclusion criteria including maternal age, type of ART, and infertility diagnosis among others. Differences in the study populations can also account for the high heterogeneity. Our review included studies from different income countries. The rates of caesarean section differ among these countries, with high-income countries, showing increased rates during the past three decades [54]. The type of health care system (public, private) is also associated with caesarean section rates, with private health systems cited as the most important structural factor in increased caesarean delivery [55, 56]. These same factors are associated with access to ART, with documented widespread disparities in access to ART between countries, and between private and public health care systems [57]. In addition, our analysis included only observational studies and not randomized clinical trials (to our knowledge inexistent in this context) which may negatively influence the quality of the evidence. However, the large sample size of our pooled analysis and long observation periods overcome these limitations.

### Comparison with existing literature

These results are consistent with the findings of three past systematic reviews and meta-analyses which examined obstetric and perinatal outcomes among the IVF/ICSI population compared to spontaneous conceptions [4, 11, 12]. Pandey et al. (2012) reported that the relative risk of having a caesarean section was 1.56 (95% CI 1.51–1.60) in IVF/ICSI conceptions compared to spontaneous conceptions [4]. They also reported a statistically increased risk of caesarean section in singleton frozen embryo transfer pregnancies compared with singletons from spontaneous conception with a relative risk ratio of 1.76 (95% CI 1.65–1.87) [4]. However, they did not evaluate and present findings on the caesarean section rates based on fertilization mode (IVF or ICSI), or other fresh embryo transfer. Helmerhorst et al. (2004) reported that rates of caesarean section were significantly higher after ART compared to spontaneous conception, with a relative risk ratio of 1.54 (95% CI 1.44–1.66) in singleton matched births [12]. The findings of these two systematic reviews support the results in this study which exhibited that there is an increased risk for caesarean section in singleton IVF/ICSI populations and frozen embryo transfer populations compared to spontaneous conception groups. Additionally, our results are similar to a meta-analysis conducted by Jackson et al. (2004) reporting a 2.13-fold increased risk of caesarean delivery among the IVF/ICSI population (OR = 2.13, 95% CI 1.72, 2.63) [11]. They also reported a 1.92-fold increased risk of elective caesarean section (OR = 1.92, 95% CI 1.49, 2.48) and a 1.47-fold increased risk of emergent caesarean section (OR = 1.47, 95% CI 1.09, 1.98) among the IVF/ICSI population compared to the spontaneous conception group [11]. However, Helmerhorst et al. (2004) and Jackson et al. (2004) did not analyze and present findings on the caesarean section rates based on fertilization mode (IVF or ICSI), or by fresh or frozen embryo transfer.

### Interpretation

Pregnancies following ART have a higher risk of adverse maternal and neonatal outcomes, which can explain the higher rate of emergent caesarean sections compared to spontaneous conceptions [58, 59]. However, provider or patient factors associated with a higher rate of elective caesarean section in ART pregnancies need to be further investigated.

## Conclusions and implications

The probability of singleton pregnancies ending in delivery by caesarean section is higher in women who conceive using ART compared to spontaneous conceptions. As access to ART has increased worldwide, there is a need to determine why caesarean sections are more common following ART than in spontaneous conceptions, and how these rates can be decreased. While the rate of caesarean section is one important health quality measure, maternal satisfaction and choice, as well as local resources and guidelines are other considerations in choosing mode of delivery. These factors were not considered in the present review. Future quantitative and qualitative studies need to address both provider and patient beliefs and preferences to offer further insight on the drivers of these findings. Preconception and pregnancy care plans following ART should focus on minimizing the risks that may lead to emergency caesarean sections. Furthermore, effective knowledge translation interventions are needed at different levels (organizational, providers, and patients) to decrease elective caesarean sections in pregnancies conceived by ART [60].

## Supplementary Information

**Additional file 1.** Search strategy using the OVID platform. A description of the search terms and results for the literature search of the MEDLINE, EMBASE, and CINHAL databases on the OVID Platform.

**Additional file 2.** Newcastle-Ottawa Scale for quality assessment and publication bias. A description of the assigned NOS scores for each of the included studies. A display of the Funnel Plots conducted to assess publication bias.

**Additional file 3.** Full text articles excluded, with reasons. A list of all full text articles removed from the systematic review and meta-analysis with the reasons for removal.

**Additional file 4.** GRADE summary of quality of evidence. GRADE approach (Grading of Recommendations Assessment, Development, and Evaluation)

## Data Availability

The datasets supporting the conclusions of this article are included within the article and its additional files.

## Abbreviations

ART: Assisted reproductive Technologies
IVF: In vitro fertilization
ICSI: Intracytoplasmic sperm injections
CINAHL: Cumulative index to nursing and allied health literature
PRISMA: Preferred Reporting Items for Systematic Reviews and Meta-analyses
PICOS: Population-Intervention- Comparators-Outcomes-Study design
IUI: Intrauterine insemination
NOS: Newcastle Ottawa Scale
OR: Odds ratio

## References

[1] National Institute of Child Health and Human Development (2019). How common is infertility?.

[2] World Health Organization (2019). Infertility definitions and terminology.

[3] Velez MP, Hamel C, Hutton B, Gaudet L, Walker M, Thuku M (2019). Care plans for women pregnant using assisted reproductive technologies: a systematic review. Reprod Health.

[4] Pandey S, Shetty A, Hamilton M, Bhattacharya S, Maheshwari A (2012). Obstetric and perinatal outcomes in singleton pregnancies resulting from IVF/ICSI: a systematic review and meta-analysis. Hum Reprod Update.

[5] Betran A, Torloni M, Zhang J, Gülmezoglu A, Section tWWGoC (2016). WHO statement on caesarean section rates. BJOG.

[6] Boerma T, Ronsmans C, Melesse DY, Barros AJD, Barros FC, Juan L (2018). Global epidemiology of use of and disparities in caesarean sections. Lancet.

[7] Maheshwari A, Pandey S, Shetty A, Hamilton M, Bhattacharya S (2012). Obstetric and perinatal outcomes in singleton pregnancies resulting from the transfer of frozen thawed versus fresh embryos generated through in vitro fertilization treatment: a systematic review and meta-analysis. Fertil Steril.

[8] Jeve YB, Potdar N, Opoku A, Khare M (2016). Donor oocyte conception and pregnancy complications: a systematic review and meta-analysis. BJOG..

[9] Qin JB, Wang H, Sheng X, Xie Q, Gao S (2016). Assisted reproductive technology and risk of adverse obstetric outcomes in dichorionic twin pregnancies: a systematic review and meta-analysis. Fertil Steril.

[10] McDonald S, Murphy K, Beyene J, Ohlsson A (2005). Perinatal outcomes of in vitro fertilization twins: a systematic review and meta-analyses. Am J Obstet Gynecol.

[11] Jackson RA, Gibson KA, Wu YW, Croughan MS (2004). Perinatal outcomes in singletons following in vitro fertilization: a meta-analysis. Obstet Gynecol.

[12] Helmerhorst FM, Perquin DAM, Donker D, Keirse MJNC (2004). Perinatal outcome of singletons and twins after assisted conception: a systematic review of controlled studies. BMJ.

[13] Moher D, Liberati A, Tetzlaff J, Altman DG (2009). Preferred reporting items for systematic reviews and meta-analyses: the PRISMA statement. J Clin Epidemiol.

[14] Lucas DN, Yentis SM, Kinsella SM, Holdcroft A, May AE, Wee M (2000). Urgency of caesarean section: a new classification. J R Soc Med.

[15] Wells GA, Shea B, O'Connell D, Peterson J, Welch V, Losos M (2009). The Newcastle-Ottawa scale (NOS) for assessing the quality if nonrandomized studies in meta-analyses.

[16] Higgins JPT, Thomas J, Chandler J, Cumpston M, Li T, Page MJ (2020). Cochrane handbook for systematic reviews of interventions version 6.1 (updated September 2020): Cochrane.

[17] Schünemann H, Brozek J, Guyatt G, Oxman A (2011). GRADE handbook for grading quality of evidence and strength of recommendation. Version 3.6.

[18] Fedder J, Loft A, Parner ET, Rasmussen S, Pinborg A (2013). Neonatal outcome and congenital malformations in children born after ICSI with testicular or epididymal sperm: a controlled national cohort study. Hum Reprod.

[19] Katalinic A, Rosch C, Ludwig M (2004). Pregnancy course and outcome after intracytoplasmic sperm injection: a controlled, prospective cohort study. Fertil Steril.

[20] Koudstaal J, Braat DD, Bruinse HW, Naaktgeboren N, Vermeiden JPW, Visser GHA (2000). Obstetric outcome of singleton pregnancies after IVF: a matched control study in four Dutch university hospitals. Hum Reprod.

[21] Olivennes F, Rufat P, Andre B, Pourade A, Quiros MC, Frydman R (1993). The increased risk of complication observed in singleton pregnancies resulting from in-vitro fertilization (IVF) does not seem to be related to the IVF method itself. Hum Reprod.

[22] Shevell T, Malone FD, Vidaver J, Porter TF, Luthy DA, Comstock CH (2005). Assisted reproductive technology and pregnancy outcome. Obstet Gynecol.

[23] Wennerholm UB, Henningsen AA, Romundstad LB, Bergh C, Pinborg A, Skjaerven R (2013). Perinatal outcomes of children born after frozen-thawed embryo transfer: a Nordic cohort study from the CoNARTaS group. Hum Reprod.

[24] Källén B, Finnström O, Nygren KG, Otterblad Olausson P, Wennerholm UB. In vitro fertilisation in Sweden: obstetric characteristics, maternal morbidity and mortality. BJOG. 2005;112(11):1529-35. 10.1111/j.1471-0528.2005.00745.x 10.1111/j.1471-0528.2005.00745.x 16225574

[25] Wennerholm UB, Hamberger L, Nilsson L, Wennergren M, Wikland M, Bergh C. Obstetric and perinatal outcome of children conceived from cryopreserved embryos. Hum Reprod. 1997;12(8):1819-25. 10.1093/humrep/12.8.1819. 10.1093/humrep/12.8.1819 9308820

[26] Anzola AB, Pauly V, Riviere O, Sambuc R, Boyer P, Vendittelli F (2019). Birthweight of IVF children is still a current issue and still related to maternal factors. Reprod Biomed Online.

[27] Apantaku O, Chandrasekaran I, Bentick B (2008). Obstetric outcome of singleton pregnancies achieved with in vitro fertilisation and intracytoplasmic sperm injection: experience from a district general hospital. J Obstet Gynaecol.

[28] Buckett WM, Chian R-C, Holzer H, Dean N, Usher R, Tan SL (2007). Obstetric outcomes and congenital abnormalities after in vitro maturation, in vitro fertilization, and intracytoplasmic sperm injection. Obstet Gynecol.

[29] D'Souza SW, Rivlin E, Cadman J, Richards B, Buck P, Lieberman BA (1997). Children conceived by in vitro fertilisation after fresh embryo transfer. Arch Dis Child Fetal Neonatal Ed.

[30] Ensing S, Abu-Hanna A, Roseboom TJ, Repping S, Van Der Veen F, Mol BWJ (2015). Risk of poor neonatal outcome at term after medically assisted reproduction: a propensity score-matched study. Fertil Steril.

[31] Gillet E, Martens E, Martens G, Cammu H (2011). Prelabour caesarean section following IVF/ICSI in older-term nulliparous women: too precious to push?. J Pregnancy.

[32] Ochsenkuhn R, Strowitzki T, Gurtner M, Strauss A, Schulze A, Hepp H (2003). Pregnancy complications, obstetric risks, and neonatal outcome in singleton and twin pregnancies after GIFT and IVF. Arch Gynecol Obstet.

[33] Olson CK, Keppler-Noreuil KM, Romitti PA, Budelier WT, Ryan G, Sparks AET (2005). In vitro fertilization is associated with an increase in major birth defects. Fertil Steril.

[34] Perri T, Chen R, Yoeli R, Merlob P, Orvieto R, Shalev Y (2001). Are singleton assisted reproductive technology pregnancies at risk of prematurity?. J Assist Reprod Genet.

[35] Pinborg A, Loft A, Henningsen AA, Rasmussen S, Andersen AN (2010). Infant outcome of 957 singletons born after frozen embryo replacement: the Danish National Cohort Study 1995-2006. Fertil Steril.

[36] Poikkeus P, Gissler M, Unkila-Kallio L, Hyden-Granskog C, Tiitinen A (2007). Obstetric and neonatal outcome after single embryo transfer. Hum Reprod.

[37] Stojnic J, Radunovic N, Jeremic K, Kotlica BK, Mitrovic M, Tulic I (2013). Perinatal outcome of singleton pregnancies following in vitro fertilization. Clin Exp Obstet Gynecol.

[38] Sun L-M, Lanes A, Kingdom CH, Kramer M, Wen SW (2014). Intrapartum interventions for singleton pregnancies arising from assisted reproductive technologies. J Obstet Gynaecol Can.

[39] Tomic V, Tomic J (2011). Neonatal outcome of IVF singletons versus naturally conceived in women aged 35 years and over. Arch Gynecol Obstet.

[40] Beyer DA, Amari F (2016). Maternal risk factors and neonatal outcomes after ART treatment - a German monocenter experience. Middle East Fertil Soc J.

[41] Carbillon L, Gronier H, Cedrin-Durnerin I, Pharisien I, Nguyen T, Valensi P (2017). The impact of ovulation induction and ovarian stimulation on the risk of pregnacy-induced hypertension and on neonatal outcomes: a case/control study. Eur J Obstet Gynecol Reprod Biol.

[42] Dayan N, Fell DB, Guo Y, Wang H, Velez MP, Spitzer K (2018). Severe maternal morbidity in women with high BMI in IVF and unassisted singleton pregnancies. Hum Reprod.

[43] Ernstad EG, Bergh C, Khatibi A, Kallen B, Westlander G, Nilsson S (2016). Neonatal and maternal outcome after blastocyst transfer: a population-based registry study. Am J Obstet Gynecol.

[44] Farhi A, Reichman B, Boyko V, Hourvitz A, Ron-El R, Lerner-Geva L (2013). Maternal and neonatal health outcomes following assisted reproduction. Reprod Biomed Online.

[45] Gambadauro P, Iliadis S, Brann E, Skalkidou A (2017). Conception by means of in vitro fertilization is not associated with maternal depressive symptoms during pregnancy or postpartum. Fertil Steril.

[46] Harlev A, Walfisch A, Oran E, Har-Vardi I, Friger M, Lunenfeld E (2018). The effect of fertility treatment on adverse perinatal outcomes in women aged at least 40 years. Int J Gynaecol Obstet.

[47] Malchau SS, Loft A, Henningsen A-KA, Nyboe Andersen A, Pinborg A (2014). Perinatal outcomes in 6,338 singletons born after intrauterine insemination in Denmark, 2007 to 2012: the influence of ovarian stimulation. Fertil Steril.

[48] Rahu K, Allvee K, Karro H, Rahu M (2019). Singleton pregnancies after in vitro fertilization in Estonia: a register-based study of complications and adverse outcomes in relation to the maternal socio-demographic background. BMC Pregnancy Childbirth.

[49] Romundstad LB, Romundstad PR, Sunde A, von During V, Skjaerven R, Gunnell D (2008). Effects of technology or maternal factors on perinatal outcome after assisted fertilisation: a population-based cohort study. Lancet.

[50] Sazonova A, Kallen K, Thurin-Kjellberg A, Wennerholm UB, Bergh C (2012). Obstetric outcome in singletons after in-vitro fertilization with cryopreserved/thawed embryos. Hum Reprod.

[51] Suzuki S, Miyake H (2007). Obstetric outcomes of elderly primiparous singleton pregnancies conceived by in vitro fertilization compared with those conceived spontaneously. Reprod Med Biol.

[52] Toshimitsu M, Nagamatsu T, Nagasaka T, Iwasawa-Kawai Y, Komatsu A, Yamashita T (2014). Increased risk of pregnancy-induced hypertension and operative delivery after conception induced by in vitro fertilization/intracytoplasmic sperm injection in women aged 40 years and older. Fertil Steril.

[53] Liu J, Linara E, Zhao W, Ma H, Ahuja K, Wang J (2015). Neonatal and obstetric outcomes of in vitro fertilization (IVF) and natural conception at a Chinese reproductive unit. Clin Exp Obstet Gynecol.

[54] Vogel JP, Betran AP, Vindevoghel N, Souza JP, Torloni MR, Zhang J (2015). Use of the Robson classification to assess caesarean section trends in 21 countries: a secondary analysis of two WHO multicountry surveys. Lancet Glob Health.

[55] Bhatia M, Banerjee K, Dixit P, Dwivedi LK (2020). Assessment of variation in cesarean delivery rates between public and private health facilities in India from 2005 to 2016. JAMA Netw Open.

[56] Sk R (2020). Does delivery in private hospitals contribute largely to caesarean section births? A path analysis using generalised structural equation modelling. PLoS One.

[57] Chambers GM, Sullivan EA, Ishihara O, Chapman MG, Adamson GD (2009). The economic impact of assisted reproductive technology: a review of selected developed countries. Fertil Steril.

[58] Elias FTS, Weber-Adrian D, Pudwell J, Carter J, Walker M, Gaudet L (2020). Neonatal outcomes in singleton pregnancies conceived by fresh or frozen embryo transfer compared to spontaneous conceptions: a systematic review and meta-analysis. Arch Gynecol Obstet.

[59] Dayan N, Joseph KS, Fell D, Laskin C, Basso O, Park A (2019). Infertility treatment and risk of severe maternal morbidity: a propensity score- matched cohort study. Can Med Assoc J.

[60] Bermudez-Tamayo C, Fernandez Ruiz E, Pastor Moreno G, Maroto-Navarro G, Garcia-Mochon L, Perez-Ramos FJ (2017). Barriers and enablers in the implementation of a program to reduce cesarean deliveries. Reprod Health.

