# Supplementary material for: Caesarean section in pregnancies conceived by assisted reproductive technology: a systematic review and meta-analysis
Source: BMC Pregnancy Childbirth. 2021 Mar 22;21:244. doi: 10.1186/s12884-021-03711-x (PMC7986269; doi:10.1186/s12884-021-03711-x)
Supplement: Supplementary file 1 — Additional file 1. Search strategy using the OVID platform. A description of the search terms and results for the literature search of the MEDLINE, EMBASE, and CINHAL databases on the OVID Platform. [file 12884_2021_3711_MOESM1_ESM.docx]

Search Strategy using the OVID Platform

| **EMBASE Database Search** | | |
| --- | --- | --- |
| **Number** | **Search** | **Results** |
| 1 | In vitro fertilization/or intracytoplasmic sperm injection/ | 26450 |
| 2 | In vitro fertilization*.mp. | 33065 |
| 3 | 1 or 2 | 47874 |
| 4 | Cesarean section/ or repeat cesarean section/ | 100754 |
| 5 | Cesarean section*.mp. | 107275 |
| 6 | Ceasarean section*.mp. | 151 |
| 7 | Caesarean section*.mp. | 31642 |
| 8 | C-section*.mp. | 3520 |
| 9 | 4 or 5 or 6 or 7 or 8 | 113419 |
| 10 | 3 and 9 | 1282 |
| 11 | Limit 10 to (human and yr= “1995-Current”) | 1186 |

| **CINAHL Database Search** | | |
| --- | --- | --- |
| **Number** | **Search** | **Results** |
| 1 | (MH “Fertilization in Vitro”) | 5702 |
| 2 | “fertilization in vitro” | 5707 |
| 3 | “in vitro fertilization” | 4886 |
| 4 | 1 or 2 or 3 | 6730 |
| 5 | (MH “Cesarean Section, Elective”) OR (MH “Cesarean Section”) OR (MH “Cesarean Section, Repeat”) | 16,700 |
| 6 | “ceasarean section” | 8 |
| 7 | “cesarean section” | 18726 |
| 8 | “c-section or cesarean section or caesarean section or cesarean delivery or caesarean or cesarean” | 6329 |
| 9 | 5 or 6 or 7 or 8 | 18786 |
| 10 | 4 and 9 | 142 |
| 11 | 4 and 9 (Limiters- Published Date: 19950101-20201231 | 141 |
| 12 | 4 and 9 (Limiters- Published Date: 19950101-20201231; Human | 59 |

| **MEDLINE Database Search** | | |
| --- | --- | --- |
| **Number** | **Search** | **Results** |
| 1 | In vitro fertilization/or intracytoplasmic sperm injection/ | 34913 |
| 2 | In vitro fertilization*.mp. | 21586 |
| 3 | 1 or 2 | 42253 |
| 4 | Cesarean section/ or repeat cesarean section/ | 44258 |
| 5 | Cesarean section*.mp. | 55993 |
| 6 | Ceasarean section*.mp. | 17778 |
| 7 | Caesarean section*.mp. | 45 |
| 8 | C-section*.mp. | 1308 |
| 9 | 4 or 5 or 6 or 7 or 8 | 64718 |
| 10 | 3 and 9 | 633 |
| 11 | Limit 10 to (human and yr= “1995-Current”) | 505 |
