# Supplementary material for: Caesarean section in pregnancies conceived by assisted reproductive technology: a systematic review and meta-analysis
Source: BMC Pregnancy Childbirth. 2021 Mar 22;21:244. doi: 10.1186/s12884-021-03711-x (PMC7986269; doi:10.1186/s12884-021-03711-x)
Supplement: Supplementary file 2 — Additional file 2. Newcastle-Ottawa Scale for quality assessment and publication bias. A description of the assigned NOS scores for each of the included studies. A display of the Funnel Plots conducted to assess publication bias. [file 12884_2021_3711_MOESM2_ESM.docx]

Newcastle-Ottawa Scale for Quality Assessment and Funnel Plot Analysis

**Table 1. Newcastle Ottawa Assessment**

Studies were either assigned a score of 0 or 1 for each of the 9 categories.

| Author, year | Selection | | | | Comparability of cohorts  ( design or analysis) | | Outcome | | | Total Score |
| --- | --- | --- | --- | --- | --- | --- | --- | --- | --- | --- |
|  | **Representativeness of the exposed cohort** | **Selection of non-exposed** | **Ascertainment of exposure** | **Presence of outcome at start of study**  0: outcome present  1: outcome not present | **control selected (the most important factor)** | **control selected (any additional factors)** | **Assessment of outcome** | **Long enough follow-up**  0: no  1: yes | **Adequacy of follow-up**  0: no  1: yes |  |
| Anzola et al. (2019) | **Score: 1**  Cohort composed of singleton IVF pregnancies delivered at a hospital in the South of France | **Score: 1**  Data on SC retrieved from databank (French AUDIPOG)  Control group was matched 1:3 considering exact age of birth, maternal age, parity, sex of neonate | **Score: 1**  Data was retrieved from secure records: Medical records and AUDIPOG network | **Score: 1** | **Score: 1**  Investigators controlled for age of birth | **Score: 1**  Investigators also controlled for maternal age, parity, and sex of neonate | **Score: 0**  20% of data missing was imputed by some variables | **Score: 1** | **Score: 1** | 8 |
| Apantaku et. al (2008) | **Score: 1**  Cohort created from case notes review of all singleton pregnancies resulting in babies weighing 500 g, achieved with the aid of IVF or ICSI at the Fertility Clinic between September 1999 and March 2004 | **Score: 0**  Controls were selected from the birth registers on the consultant maternity unit and the midwife-led unit with the following criteria: spontaneous conception, maternal age (tolerance +2 years), parity and singleton pregnancy | **Score: 1**  Data was retrieved from secure records: retrospectively reviewed case notes from a fertility clinic | **Score: 1** | **Score: 1**  Investigators controlled for age | **Score: 1**  Investigators also controlled for parity | **Score: 1**  Record linkage | **Score: 1** | **Score: 1**  Retrospective review | 8 |
| Beyer et al. (2016) | **Score: 1**  Region is rural in majority with a predominantly Caucasian population (exposed group is somewhat representative of the average ART user in the community) | **Score: 1**  Data extracted from electronic database of the center, therefore it is drawn from the same community as the exposed cohort | **Score: 1**  Data retrieved from secure records: center database | **Score: 1** | **Score: 0** Analysis of interest is unadjusted | **Score: 0**  Analysis of interest is unadjusted | **Score: 1**  Data retrieved from database therefore record linkage | **Score: 1** | **Score: 1** | 7 |
| D'Souza et al. (1997) | **Score: 1**  Cohort individuals retrieved from the IVF Unit at St Mary’s Hospital, Manchester | **Score: 1**  Non-exposed individuals are drawn from the same community as exposed cohort | **Score: 1**  Data retrieved from secure records: case notes | **Score: 1** | **Score: 1**  Investigators matched for age | **Score: 1**  Investigators also matched for sex and social class | **Score: 1**  Data retrieved from case notes therefore record linkage | **Score: 1** | **Score: 1** | 9 |
| Dayan et al. (2018) | **Score: 1**  Data source was the Canadian Assisted Reproductive Technologies Register (CARTR Plus) linked with the Ontario birth registry (BORN Information System) | **Score: 1**  Women who delivered a live or stillborn infant weighing ≥500 g at ≥20 weeks gestation were eligible for inclusion in the cohort if they conceived within the 1-year study period | **Score: 1**  Data retrieved from secure records: CARTR and BORN database | **Score: 1** | **Score: 1**  Investigators matched for age | **Score: 1**  Investigators also matched for parity, education, income, and baseline maternal comorbidity | **Score: 1**  Data retrieved from database therefore record linkage | **Score: 1** | **Score: 1** | 9 |
| Ensing et al.(2015) | **Score: 1**  Data source was the Netherlands Perinatal Registry (PRN) which covers about 96% of all deliveries in the Netherlands and is a linkage of : the midwifery registry (LVR1), the obstetrics registry (LVR2), and the neonatology registry (LNR) of hospital admissions of newborns | **Score: 1**  Data from the PRN with one-to-one matching without replacement on the closest propensity score of the MAR women and SC women | **Score: 1**  Data retrieved from secure records:  PRN | **Score: 1** | **Score: 1** Investigators matched for maternal age | **Score: 1**  Investigators also matched for parity, previous c-section, level of car at onset of labor, maternal ethnicity, socioeconomic status, diabetes mellitus, hypertensive disease, gestational age, birth weight, congenital anomalies, fetal presentation, and mode of delivery | **Score: 1**  Data retrieved from database therefore record linkage | **Score: 1** | **Score: 1** | 9 |
| Gillet et al. (2011) | **Score: 1**  Data retrieved Flemisch Centre for Perinatal Epidemiology (SPE- Studiecentrum voor Perinatale Epidemiologie) where official perinatal forms for each birth over 0.5g in the surronding region is sent | **Score: 1**  Non-exposed individuals are drawn from the same database as the exposed cohort | **Score: 1**  Data retrieved from secure records at Flemisch Centre for Perinatal Epidemiology (SPE) | **Score: 1** | **Score: 1**  Investigators matched for age | **Score: 1**  Investigators also matched for parity | **Score: 1**  Data retrieved from database therefore record linkage | **Score: 1** | **Score: 1** | 9 |
| Harlev et al. (2018) | **Score: 1**  Cohort consisted of all women aged at least 40 years who had deliveries of singleton neonates at the Soroka University Medical Center, Beer Sheva, Israel | **Score: 1**  Non-exposed individuals are drawn from the same community | **Score: 1**  Patient data retrieved from the institution’s perinatal electronic database | **Score: 1** | **Score: 1**  Investigators controlled for maternal age | **Score: 1**  Investigators also controlled for ethnicity, diabetes mellitus, pre-eclampsia, gravidity, and type of ART | **Score: 1**  Data retrieved from database therefore record linkage | **Score: 1** | **Score: 1** | 9 |
| Perri et al. (2001) | **Score: 1**  Cohort is somewhat representative of average ART in community with the data being retrieved from a medical center database | **Score: 1**  All data were prospectively collected on a computerized database | **Score: 1**  Data retrieved from secure records at the Rabin Medical centre | **Score: 1** | **Score: 1**  Investigators matched for maternal age | **Score: 1**  Investigators also matched for parity, ethnic origin, and gravidity | **Score: 1**  Data retrieved from database therefore record linkage | **Score: 1** | **Score: 1** | 9 |
| Poikkeus et al. (2007) | **Score: 1**  Cohort pregnancies were from the infertility clinic of Helsinki university central hospital (HUCH) and pregnancies were linked to the Finnish Medical Birth Register (MBR), Hospital Discharge Register (HDR) and the Register of Congenital Malformations (RCM) | **Score: 0**  Non-exposed individuals are drawn from a different community | **Score: 1**  Data retrieved from secure records from the Finnish MBR, HDR and the RCM | **Score: 1** | **Score: 1**  Investigators adjusted for maternal age | **Score: 1**  Investigators also adjusted for parity and socioeconomic status | **Score: 1**  Data retrieved from databases (HUCH, MDR, RCM) therefore record linkage | **Score: 1** | **Score: 1** | 8 |
| Romundstad et al. (2008) | **Score: 1**  Cohort data retrieved from a medical birth registry (MBR) | **Score: 1**  Non-exposed individuals are drawn from the same community (MBR) | **Score: 1**  Data retrieved from secure records from MBR | **Score: 1** | **Score: 1**  Investigators adjusted for maternal age | **Score: 1**  Investigators also adjusted for parity, off-spring sex, time between pregnancies, and year of delivery | **Score: 1**  Data retrieved from registry therefore record linkage | **Score: 1** | **Score: 1** | 9 |
| Suzuki et al. (2007) | **Score: 1**  Data retrieved from Japanese Red Cross Katsushika Maternity Hospital | **Score: 1**  Non-exposed individuals are drawn from the same community | **Score: 1**  Data retrieved from secure hospital records | **Score: 1** | **Score: 1** Investigators adjusted for maternal age | **Score: 0**  No explicit statement on additional adjustments or matching | **Score: 1**  Data retrieved from secure records therefore record linkage | **Score: 1** | **Score: 1** | 8 |
| Fedder et al. (2012) | **Score: 1**  Exposed groups were identified by a cross-linkage of the Danish IVF register and the Danish Medical Birth Register (MBR) | **Score: 1**  Non-exposed individuals were identified using MBR | **Score: 1**  Data retrieved from secure records: Danish IVF register and MBR | **Score: 1** | **Score: 1**  Investigators adjusted for maternal age | **Score: 1**  Investigators also adjusted for parity, child, gender, and infant year of birth | **Score: 1**  Data retrieved from database therefore record linkage | **Score: 1** | **Score: 1** | 9 |
| Gambadauro et al. (2017) | **Score: 1**  Data retrieved from Uppsala University Hospital specifically from the (BASIC (Biology, Affect, Stress, Imaging, Cognition) project, a population-based longitudinal study | **Score: 1**  Non-exposed individuals drawn from same community (BASIC project) | **Score: 0**  Data retrieved from a web-based self-administered structured questionnaire | **Score: 1** | **Score: 1** Investigators adjusted for age | **Score: 1**  Investigators also adjusted for parity, BMI, education, depression history, and Stressful life events (SLEs) | **Score: 0**  Data retrieved from self-report with no further description | **Score: 1** | **Score: 1** | 7 |
| Sun et al. (2014) | **Score: 1**  Data extracted from BORN database | **Score: 1**  Non-exposed individuals are drawn from the same community (BORN database) | **Score: 1**  Data retrieved from secure records: BORN database | **Score: 1** | **Score: 1** Investigators matched for maternal age | **Score: 1**  Investigators also matched for parity | **Score: 1**  Data retrieved from database therefore record linkage | **Score: 1** | **Score: 1** | 9 |
| Malchau et al. (2014) | **Score: 1**  Data for exposed group retrieved from Danish ART register and Danish medical birth register (MBR) | **Score: 1**  Non-exposed individuals were drawn from the same community (MBR) | **Score: 1**  Data retrieved from secure records:  The Danish ART Register, MBR, and hospital discharge register | **Score: 1** | **Score: 1** Investigators adjusted for maternal age | **Score: 1**  Investigators also adjusted for parity, child gender, year of birth, smoking, maternal BMI, elective cesarean section and induction of labor | **Score: 1**  Data retrieved from registers therefore record linkage | **Score: 1** | **Score: 1** | 9 |
| Buckett et al. (2007) | **Score: 1**  Cohort consisted of all pregnancies delivered at McGill after IVF with a birth weight of at least 500g from 1998-2003 | **Score: 1**  Non-exposed individuals were drawn from the same community with 1 to 1 matched controls selected from same database | **Score: 1**  Data retrieved from secure records: McGill Obstetric and Neonatal Database | **Score: 1** | **Score: 1**  Investigators matched for age | **Score: 1**  Investigators matched for parity | **Score: 1**  Data retrieved from database therefore record linkage | **Score: 1** | **Score: 1** | 9 |
| Farhi et al. (2013) | **Score: 1**  Cohort composed after prospective recruitment from 2 IVF units | **Score: 0**  Non-exposed individuals were recruited differently, with a mixture of prospective and retrospective recruitment and from different communities | **Score: 0**  No explicit statement on the data source | **Score: 1** | **Score: 1** Investigator adjusted for maternal age (in logistic regression) | **Score: 1**  Investigator also adjusted for education (in logistic regression) and controlled for obstetric history, pre-existing hypertension, pre-existing diabetes, BMI and smoking | **Score: 0**  Data retrieved from participant interview (self-report) | **Score: 1** | **Score: 1**  loss to follow up appears to be <10% | 6 |
| Liu et al. (2015) | **Score: 1**  Cohort consisted of all women pregnant through IVF at their center | **Score: 0**  Non-exposed individuals were randomly selected from the SC population at the same center  Used active consent, no statement about how many were approached vs how many were consented | **Score: 0**  No explicit statement on the data source | **Score: 1** | **Score: 1**  Investigators stratified by maternal age | **Score: 0**  Investigators did not control for other factors | **Score: 0**  No explicit statement on the data source | **Score: 1** | **Score: 0**  No statement regarding loss to follow up | 4 |
| Rahu et al. (2019) | **Score: 1**  All data of the liveborn singletons to primiparas women aged 25–40 years was obtained from the Estonian Medical Birth Registry (MBR) | **Score: 1**  Non-exposed individuals were selected from the same database (Estonian MBR) | **Score: 1**  Data retrieved from secure records: The Estonian Medical Birth Registry | **Score: 1** | **Score: 1**  Investigators adjusted for maternal age | **Score: 1**  Investigators also adjusted for social data including race, ethnicity, and economic status | **Score: 1**  Data retrieved from database therefore record linkage | **Score: 1** | **Score: 1** | 9 |
| Stojnic et al. (2013) | **Score: 1**  Cohort consisted of consecutive singleton pregnancies after ART at a single center in Serbia from 2006-2010 (oocyte donation, reduced twins and frozen embryos were excluded) | **Score: 1**  Non-exposed individuals were drawn from the same community | **Score: 1**  Data retrieved from secure records including chart review and from participant questionnaire | **Score: 1** | **Score: 1**  Investigators matched for maternal age | **Score: 1**  Investigators also matched for education, BMI, parity, time, and place | **Score: 1**  Data retrieved from medical records (record linkage) and interview | **Score: 1** | **Score: 1** | 9 |
| Tomic et al. (2011) | **Score: 1**  Cohort consisted of singleton IVF primiparas 35 years or older from a single center with birth weight >500g from 2006-2009 - fresh cycles only, reductions excluded.  (excluded oocyte donation, cryopreservation) | **Score: 1**  Non-exposed individuals were drawn from the same community | **Score: 1**  Data retrieved from secure records: hospital medical records | **Score: 1** | **Score: 1**  Investigators matched by maternal age | **Score: 1**  Investigators also matched by ethnicity, gravidity, smoking, BMI, weight gain, site and time of delivery | **Score: 1**  Data retrieved from medical records therefore record linkage | **Score: 1** | **Score: 1** | 9 |
| Toshimitsu et al. (2014) | **Score: 1**  Cohort data retrieved from obstetric records and consisted of all the pregnant women aged 40 and older who delivered singletons during the study period | **Score: 1**  Non-exposed individuals were drawn from the same community | **Score: 1**  Data retrieved from secure records: hospital medical records | **Score: 1** | **Score: 0**  No explicit mention of matching or adjustment | **Score: 0**  No explicit mention of matching or adjustment | **Score: 1**  Data retrieved from medical records therefore record linkage | **Score: 1** | **Score: 1**  Database and record linkage allowing for adequate follow-up | 7 |
| Shevell et al. (2005) | **Score: 1**  Cohort consisted of women who conceived using IVF in a prospective trial; recruited at first trimester screening | **Score: 1**  Non-exposed individuals also recruited from prospective trial at first trimester screening | **Score: 1**  Data retrieved from structured interview and chart review | **Score: 1** | **Score: 1**  Investigators conducted multivariate analysis controlling for age | **Score: 1**  Investigators also controlled for race, marital status, education, prior preterm delivery, prior pregnancy with anomaly, BMI, smoking and bleeding in pregnancy in analysis | **Score: 1**  Data retrieved from chart review and interview, therefore record linkage | **Score: 1** | **Score: 0**  Prospective recruitment but no statement about loss to follow up | 8 |
| Sazonova et al. (2012) | **Score: 1**  Cohort consisted of all IVF clinics in Sweden 2002-2006; Linked to Swedish medical birth registry | **Score: 1**  Non-exposed cohort consisted of all non-IVF pregnancies in Sweden from 2002-2006 | **Score: 1**  Data retrieved from secure records: medical records and database. | **Score: 1** | **Score: 1**  Investigators stratified by maternal age | **Score: 1**  Investigators also stratified by parity | **Score: 1**  Data retrieved from medical records and database therefore record linkage | **Score: 1** | **Score: 1**  Database and linkage very complete | 9 |
| Carbillon et al. (2017) | **Score: 1**  Cohort consisted of all ART patients from a single center in France from 2007-2010 | **Score: 1**  Non- exposed patients from the same community during the same time period | **Score: 1**  Data retrieved from secure records: hospital medical records | **Score: 1** | **Score: 0**  Outcomes of interest are unadjusted | **Score: 0**  Outcomes of interest are unadjusted | **Score: 1**  Data retrieved from medical records and database therefore record linkage | **Score: 1** | **Score: 1**  Database includes all women giving birth at the center allowing for adequate follow-up | 7 |
| Ernstad et al. (2016) | **Score: 1**  Cohort data was retrieved from all IVF clinics in Sweden from 2002-2013, all IVF singletons with autologous oocytes were included | **Score: 1**  Non-exposed individuals drawn from the same community during the same time period | **Score: 1**  Data retrieved from secure records: linkage of registries | **Score: 1** | **Score: 0**  Unadjusted analysis for outcomes of interest (Adjusted only for birth defects) | **Score: 0**  Unadjusted analysis for outcomes of interest | **Score: 1**  Data retrieved from registry therefore record linkage | **Score: 1** | **Score: 1** | 7 |
| Katalinic et al. (2004) | **Score: 1**  Cohort composed from prospective recruitment of ICSI patients from 1998-2000 from a center in Germany | **Score: 1**  Non-exposed individuals recruited from the same community between 1993-2001 using the birth registry model of recruitment | **Score: 1**  Data retrieved from a combination of chart review and structured telephone interview | **Score: 1** | **Score: 0** Unadjusted analysis for outcomes of interest | **Score: 0**  Unadjusted analysis for outcomes of interest | **Score: 1**  Data retrieved from structured interview, doctor standardized examination (exposure group), and database (control group) | **Score: 1** | **Score: 1**  84% follow up in  no statement regarding completeness of registry, but likely good coverage | 7 |
| Koudstaal et al. (2000) | **Score: 1**  Cohort composed of IVF pregnancies 16 weeks or greater at 4 centers in the Netherlands | **Score: 1**  Non-exposed individuals recruited from the same centers with matching conducted | **Score: 1**  Data retrieved from secure records: hospital medical registry | **Score: 1** | **Score: 1**  Investigators matched for age | **Score: 1**  Investigators also matched for parity, ethnicity, height, weight, smoking, obstetric history, medical history and date of delivery | **Score: 1**  Data retrieved from registry therefore record linkage | **Score: 1** | **Score: 1** | 9 |
| Ochsenkuhn et al. (2003) | **Score: 1**  Cohort consisted of all IVF pregnancies from a 5-year period (1991-1996), > 24 weeks gestational age or > 499 g at birth. With stillbirths excluded | **Score: 1**  Non-exposed individuals selected from the same center | **Score: 1**  Data retrieved from secure hospital medical records | **Score: 1** | **Score: 1**  Investigators matched for age | **Score: 1**  Investigators also matched for parity | **Score: 1**  Data retrieved from medical records therefore record linkage | **Score: 1** | **Score: 1**  Participants from a system that encompasses all births at the center | 9 |
| Olivennes et al. (1993) | **Score: 1**  Cohort consisted of IVF pregnancies at a single French center from 1987-1989 | **Score: 1**  Non-exposed individuals selected from the same center during the same time period | **Score: 1**  Data retrieved from secure records: chart review | **Score: 1** | **Score: 0**  Unadjusted analysis for outcomes of interest | **Score: 0**  Unadjusted analysis for outcomes of interest | **Score: 1**  Data retrieved from chart review therefore record linkage | **Score: 1** | **Score: 1** | 7 |
| Olson et al. (2005) | **Score: 1**  Cohort consisted of all IVF births at the University of Iowa clinic from 1989-2002 (out of state excluded; linked to birth certificates) | **Score: 1**  Non-exposed group selected in a 5 to 1 selection of a naturally conceived control from a 65-county surrounding area | **Score: 0**  Exposure to IVF determined by IVF database however, there was no explicit statement about the coverage of the IVF program | **Score: 1** | **Score: 1**  Investigators matched by maternal age | **Score: 1**  Investigators also matched by year of birth and ethnicity | **Score: 1**  Data retrieved from outcome from birth registry | **Score: 1** | **Score: 1** | 8 |
| Pinborg et al. (2010) | **Score: 1**  Danish population-based cohort; 1995-2006 (multiple types of IVF subgroups); from compulsory IVF registry | **Score: 0**  non-ART singletons from the same time-period; randomly selected at 5:1 however, IUI and OI may be in the control population | **Score: 1**  Data retrieved from secure records: medical records database | **Score: 1** | **Score: 1**  Maternal age adjusted for in logistic regression | **Score: 1**  Parity also adjusted for in logistic regression  also controlled for child gender and year of birth | **Score: 1**  Data retrieved from medical record database therefore record linkage | **Score: 1** | **Score: 1**  Registry very complete | 8 |
| Wennerholm et al. (2013) | **Score: 1**  Cohort consisted of all IVF pregnancies in a database from Denmark, Norway and Sweden; only excluded for stillbirth and missing outcome data | **Score: 1**  Non-exposed group selected from the same community in a 4:1 ratio | **Score: 1**  Data retrieved from secure records: medical records database | **Score: 1** | **Score: 1**  Investigators matched for parity | **Score: 1**  Investigators also matched for year of birth | **Score: 1**  Data retrieved from medical record database: National registries – CoNARTaS group | **Score: 1** | **Score: 1**  Yes, only included a participant if they had a birth in the system | 9 |
| MAR: Medically Assisted Reproduction SC: Spontaneous conception IVF: In-vitro fertilization ICSI: intracytoplasmic sperm injection ART: Assisted reproductive Technologies  IUI: intrauterine insemination  OI: ovulation induction | | | | | | | | | | |

Funnel plots by meta-analysis - publication bias

**Figure 1. IVF/ICSI versus spontaneous conception**

| **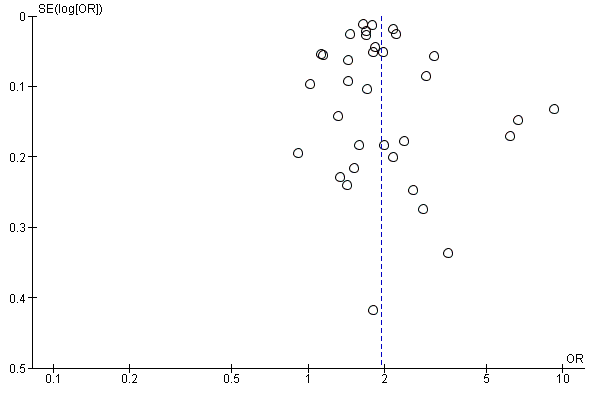** |
| --- |
| **Caesarean Section Total** |
| **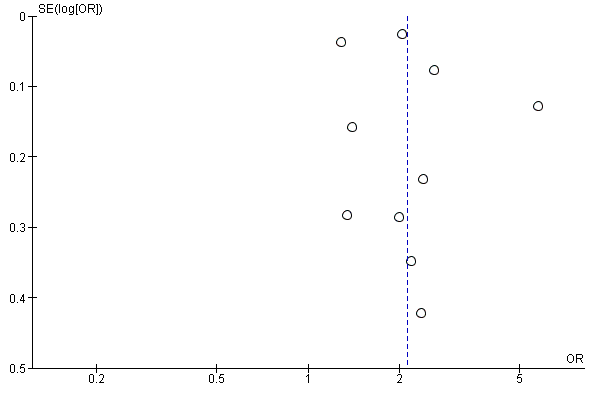** |
| **Elective Caesarean Section** |
| **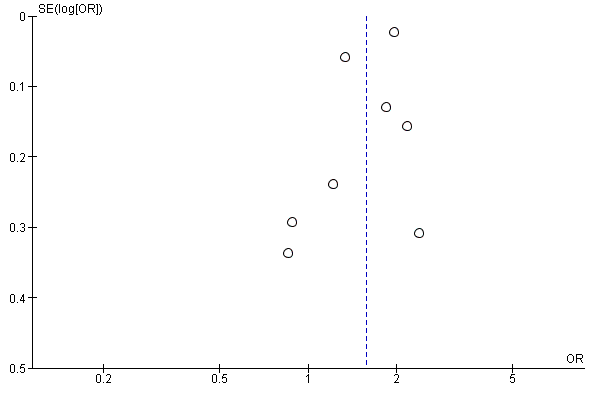** |
| **Emergent Caesarean Section** |

**Figure 2. In vitro fertilization (IVF) versus spontaneous conception**

| **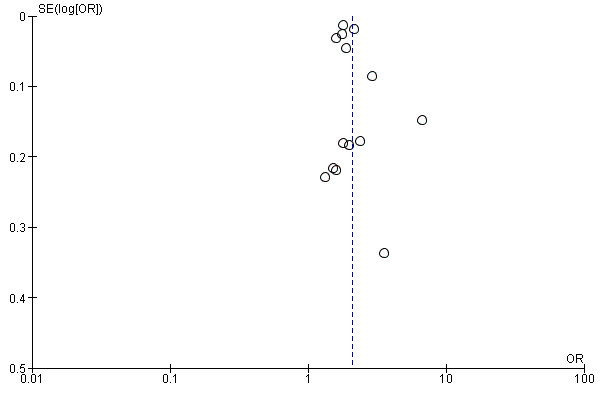** |
| --- |
| **Caesarean Section Total** |
| **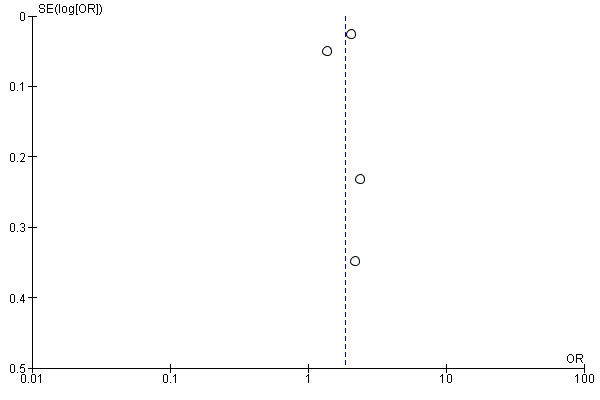** |
| **Elective Caesarean Section** |
| **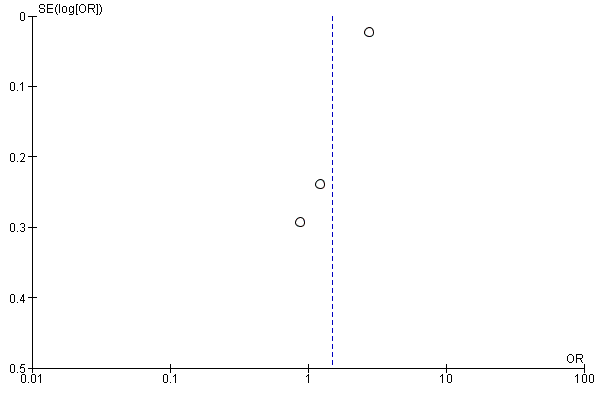** |
| **Emergent Caesarean Section** |

**Figure 3. Intracytoplasmic sperm injection (ICSI) versus spontaneous conception**

| **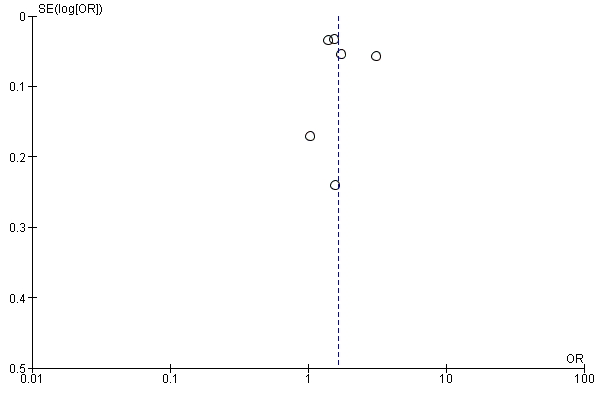** |
| --- |
| **Caesarean Section Total** |

**Figure 4. Fresh embryo transfer versus spontaneous conception**

| **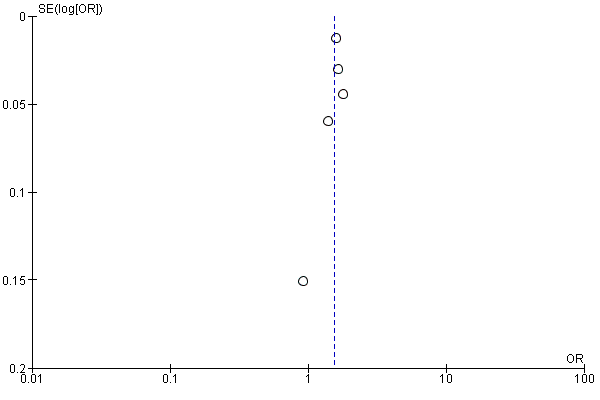** |
| --- |
| **Caesarean Section Total** |

**Figure 5. Frozen embryo transfer versus spontaneous conception**

| **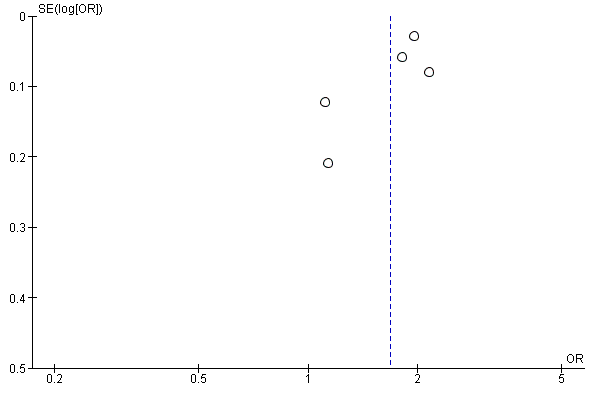** |
| --- |
| **Caesarean Section Total** |
