# Supplementary material for: Caesarean section in pregnancies conceived by assisted reproductive technology: a systematic review and meta-analysis
Source: BMC Pregnancy Childbirth. 2021 Mar 22;21:244. doi: 10.1186/s12884-021-03711-x (PMC7986269; doi:10.1186/s12884-021-03711-x)
Supplement: Supplementary file 3 — Additional file 3. Full text articles excluded, with reasons. A list of all full text articles removed from the systematic review and meta-analysis with the reasons for removal. [file 12884_2021_3711_MOESM3_ESM.docx]

**Full Text Articles Excluded, with reasons (n=88)**

Study Design: Abstract (n=26) ^1-26^

Full text not available (n=13) ^27-39^

Wrong study design (n=10) ^40-49^

Wrong Exposure (n=5) ^50-54^

Duplicate (n=4) ^55-58^

Wrong Comparator (n=4) ^59-62^

Wrong Intervention (n=4) ^63-66^

Study without spontaneous conception as control group (n=4) ^67-70^

No separation of singleton and multiple pregnancies (n=4) ^71-74^

No separation of autologous oocytes and donor oocytes (n=3) ^75-77^

Did not explicitly report on women who became pregnant with ART (n=3) ^78-80^

Excluded language (n=3) ^81-83^

Overlapped cohort (n=2) ^84,85^

IVF as a small subgroup (n=1) ^86^

No separation of invasive ART procedures from non-invasive ART procedures (n=1) ^87^

Twins or multiples (n=1) ^88^

References

1. Bannister W, Lo M. Effects of fertility treatments controlling for multiple gestation and maternal age. *Journal of Women's Health.* 2014;23(4):11.

2. Cabello Y, Ricciarelli E, Fernandez-Shaw S, et al. Perinatal outcome after assisted reproduction techniques in spain compared with the average of national standard population. *Human Reproduction.* 2012;27(SUPPL. 2).

3. Diaconu R, Stanescu L, Niculescu C, Popescu M, Selaru L. Caesarean section in oltenia county-trends and motivations-an observational study. *European Journal of Pediatrics.* 2016;175(11):1857.

4. Enache A, Marinescu B, Enache T. Parameters predicting pregnancy and fetal outcome in IVF/ICSI patients: Is it possible? *International Journal of Gynecology and Obstetrics.* 2012;119(SUPPL. 3):S340.

5. Furuya S, Yamaguchi T, Kagawa T, Takahashi H, Kubonoya K. Risk factors for abnormal umbilical cordinsertions among term singleton art pregnancies. *Fertility and Sterility.* 2016;106(Supplement 3):e178-e179.

6. Harlev A, Har-Vardi I, Lunenfeld E, et al. Is fertility treatment an additional perinatal risk factor in women over 40 years old? *Human Reproduction.* 2016;31(Supplement 1).

7. Kamisawa E, Hirabayashi N, Wakasa S, Inoue K, Yusa H. Assessment of delivery and neonatal outcomes after ART and natural conceptions in order to obtain useful information for supporting the couples with ART pregnancies. *Human Reproduction.* 2012;27(SUPPL. 2).

8. Kapustova I, Zubor P, Biskupska-Bodova K, et al. Obstetric and perinatal outcomes following in vitro fertilization. *Journal of Perinatal Medicine.* 2013;41(SUPPL. 1).

9. Kesmodel US, Ingerslev HJ, Lemmen JG, Rasmussen IA, Bay B. Adverse effects in pregnancy after treatment with preimplantation genetic diagnosis-a Danish national multicenter followup study. *Human Reproduction.* 2015;30(SUPPL. 1).

10. Luke B, Brown MB, Spector LG. Risk of severe maternal morbidity: A us study in seven states. *Fertility and Sterility.* 2017;108(3 Supplement 1):e72-e73.

11. McNamee K, Brigham S, Wood S. Obstetric outcomes following assisted reproductive technology in a district general hospital. *Human Fertility.* 2013;16(3):e68.

12. Nachajova M, Kudela E, Biringer K, Visnovsky J, Zibolen M, Danko J. Analysis of perinatal outcomes after in vitro fertilization. *Journal of Maternal-Fetal and Neonatal Medicine.* 2016;29(Supplement 1):216.

13. Nagai R, Fukunaga N, Kitasaka H, et al. Pregnancy & perinatal outcome after in vitro fertilization in 1341 cycles. *Human Reproduction.* 2011;26(SUPPL. 1).

14. Amini L, Safaie Z, Jamshidi RA, Neisani Samani L. Maternal outcomes after in vitro fertilization. *International Journal of Fertility and Sterility.* 2015;9(Supplement 1):104.

15. Amrane S, Ghosh P, Reichman DE, Rosenwaks Z, Gelber SE. Maternal and neonatal outcomes in women of advanced maternal age (AMA) undergoing two in vitro fertilization (IVF) singleton pregnancies, as compared to one IVF twin pregnancy. *Fertility and Sterility.* 2016;106(Supplement 3):e34.

16. Perez Martinez N, Suarez-Gil P, Garcia S, Llaneza-Suarez D, Sanchez SJL, Llaneza P. Perinatal results after IVF/ICSI. A prospective study. *Human Reproduction.* 2015;30(SUPPL. 1).

17. Ramos LG, Wang ET, Greene N, Vyas N, Pisarska MD. Mode of conception is not associated with adverse pregnancy outcomes. *Fertility and Sterility.* 2015;104(3 SUPPL. 1):e212.

18. Smith MB, Mandelbaum RS, Ho J, Bendikson K, Paulson RJ. Mode of delivery in gestational carrier (GC) pregnancies in women with and without a history of cesarean section (CS). *Fertility and Sterility.* 2019;112(3 Supplement):e300.

19. Sundheimer LW, Wang ET, Quant C, Spades C, Simmons CF, Pisarska MD. Adverse perinatal outcomes associated with fertility treatment in late preterm infants. *Fertility and Sterility.* 2016;106(Supplement 3):e175.

20. Szymusik I, Kosinska-Kaczynska K, Marianowski P, Wielgos M. The obstetric outcome of IVF Singletons. *Journal of Maternal-Fetal and Neonatal Medicine.* 2016;29(Supplement 1):280.

21. Taylor JS, Reiss J, Lin S, Grunebaum A. Documentation of endometriosis at time of cesarean delivery. *Journal of Minimally Invasive Gynecology.* 2012;19(6 SUPPL. 1):S98.

22. Teresa Del Moral T, Dominguez S, Kooning K, Vanburskit S. Neonatal outcomes of very premature infants born after in vitro fertilization. *Archives of Disease in Childhood.* 2012;97(SUPPL. 2):A497.

23. Tolcher MC, Magistrado L, Suhag A, Aagaard KM. 579: Obstetric and neonatal outcomes with advanced maternal age >=45 years. *American Journal of Obstetrics and Gynecology.* 2019;220(1 Supplement):S385.

24. Vakrilova L, Slavov S, Slancheva B, Hitrova S, Emilova Z. Problems and neonatal outcome of very low birth weight newborn infants after in vitro fertilization. *Journal of Maternal-Fetal and Neonatal Medicine.* 2012;25(SUPPL. 2):86.

25. Zecevic N, Rakic S, Jankovic Raznatovic S, et al. Complications and outcomes of pregnancies after ART procedures , in women with advanced age. *Journal of Perinatal Medicine.* 2015;43(SUPPL. 1).

26. Zile I, Jefremova I, Villerusa A, Gavare I. Perinatal outcomes from in vitro fertilization-retrospective cohort study in Latvia. *Journal of Maternal-Fetal and Neonatal Medicine.* 2016;29(Supplement 1):187-188.

27. Ashrafi M. Art outcomes. *International Journal of Fertility and Sterility.* 2016;10(Supplement 1):16.

28. Beyer DA, Amari F, Klett J, Diedrich K. Does the mode of delivery depend on the way of conception? *Archives of Gynecology and Obstetrics.* 2012;286(1 SUPPL. 1):S204.

29. Bielak A, Hincz P, Brot A, Wilczynski J. [Pregnancy and childbirth course after IVF-ET patients admitted to KMMP ICZMP on Lodz between 1996-2002]. *Ginekologia polska.* 2003;74(10):1049-1054.

30. Nassar S, Boutros J, Aboulghar H, Mansour R, Hussein M, Aboulghar M. Perinatal outcome after in vitro fertilization and spontaneous pregnancy: A comparative study. *Middle East Fertility Society Journal.* 1996;1(2):151-158.

31. Pacarada M, Kongjeli N, Kongjeli G, Obertinca B. Birth management after in vitro fertilisation: a report of 178 cases. *Nigerian journal of medicine : journal of the National Association of Resident Doctors of Nigeria.* 2009;18(1):29-31.

32. Rueda SSO, Delgadillo JCB, Chavez FCP, et al. Perinatal result of an institutional assisted reproduction program. *Ginecologia y Obstetricia de Mexico.* 2004;72(12):619-627.

33. Severinski NS, Mamula O, Vlastelic I. Adverse perinatal outcome after assisted reproductive technology. *Gynaecologia et Perinatologia, Supplement.* 2006;15(1):73-76.

34. Srisombut C, Rojanasakul A, Suchartwatnachai C, Choktanasiri W, Weerakiet S, Chinsomboon S. Outcome of pregnancy in IVF-ET cycle at Ramathibodi Hospital. *Journal of the Medical Association of Thailand = Chotmaihet thangphaet.* 1995;78(12):657-661.

35. Wang Q-Q, Zhu Y-M, Wu M-Y. [Health status of mothers undergoing in vitro fertilization and their offspring]. *Zhejiang da xue xue bao Yi xue ban = Journal of Zhejiang University Medical sciences.* 2009;38(5):515-520.

36. Wurfel W. Indications for caesarean section after in vitro fertilisation. *Gynakologische Praxis.* 2006;30(1):117-118.

37. Yanaihara A, Michiba F, Ohgi S, et al. Medical intervention during labor increases after in vitro fertilization pregnancy. *Clinical and Experimental Obstetrics and Gynecology.* 2017;44(6):862-865.

38. Zlopasa G. Methods of assisted reproduction - Pregnancies outcome. *Gynaecologia et Perinatologia, Supplement.* 2006;15(1):59-65.

39. Luke B, Brown MB, Wantman E, et al. Risk of prematurity and infant morbidity and mortality by maternal fertility status and plurality. *Journal of Assisted Reproduction & Genetics.* 2018;36(1):121-138.

40. Di Tommaso M, Sisti G, Colombi I, et al. Influence of assisted reproductive technologies on maternal and neonatal outcomes in early preterm deliveries. *Journal of Gynecology Obstetrics and Human Reproduction.* 2019;48(10):845-848.

41. Maman E, Lunenfeld E, Levy A, Vardi H, Potashnik G. Obstetric outcome of singleton pregnancies conceived by in vitro fertilization and ovulation induction compared with those conceived spontaneously. *Fertility and sterility.* 1998;70(2):240-245.

42. Pandey S, Shetty A, Hamilton M, Bhattacharya S, Maheshwari A. Obstetric and perinatal outcomes in singleton pregnancies resulting from IVF/ICSI: a systematic review and meta-analysis. *Human Reproduction Update.* 2012;18(5):485-503.

43. Al-Fifi S, Al-Binali A, Al-Shahrani M, et al. Congenital anomalies and other perinatal outcomes in ICSI vs. naturally conceived pregnancies: A comparative study. *Journal of Assisted Reproduction and Genetics.* 2009;26(7):377-381.

44. Allen VM, Wilson RD, Cheung A, et al. Pregnancy Outcomes After Assisted Reproductive Technology. *Journal of Obstetrics and Gynaecology Canada.* 2006;28(3):220-233.

45. Chaveeva P, Carbone IF, Syngelaki A, Akolekar R, Nicolaides KH. Contribution of method of conception on pregnancy outcome after the 11-13 weeks scan. *Fetal Diagnosis & Therapy.* 2011;30(1421-9964 (Electronic)).

46. Szymusik I, Kosinska-Kaczynska K, Krowicka M, Sep M, Marianowski P, Wielgos M. Perinatal outcome of in vitro fertilization singletons - 10 years' experience of one center. *Archives of Medical Science.* 2019;15(3):666-672.

47. Talaulikar VS, Arulkumaran S. Reproductive outcomes after assisted conception. *Obstetrical and Gynecological Survey.* 2012;67(9):566-583.

48. Verlaenen H, Cammu H, Derde MP, Amy JJ. Singleton pregnancy after in vitro fertilization: Expectations and outcome. *Obstetrics and Gynecology.* 1995;86(6):906-910.

49. Zollner U, Dietl J. Perinatal risks after IVF and ICSI. *Journal of perinatal medicine.* 2013;41(1):17-22.

50. Kozinszky Z, Zadori J, Orvos H, Katona M, Pal A, Kovacs L. Risk of cesarean section in singleton pregnancies after assisted reproductive techniques. *The Journal of reproductive medicine.* 2003;48(3):160-164.

51. Kozinszky Z, Zadori J, Orvos H, Katona M, Pal A, Kovacs L. Obstetric and neonatal risk of pregnancies after assisted reproductive technology: a matched control study. *Acta obstetricia et gynecologica Scandinavica.* 2003;82(9):850-856.

52. Norrman E, Bergh C, Wennerholm UB. Pregnancy outcome and long-term follow-up after in vitro fertilization in women with renal transplantation. *Human Reproduction.* 2015;30(1):205-213.

53. Abramov Y, Elchalal U, Schenker JG. Obstetric outcome of in vitro fertilized pregnancies complicated by severe ovarian hyperstimulation syndrome: a multicenter study. *Fertility and sterility.* 1998;70(6):1070-1076.

54. Al Inizi SAJT, Asaad M, Schick J. The outcome of in-vitro fertilization/intracytoplasmic sperm injection (IVF/ICSI) cycles complicated by moderate-severe ovarian hyperstimulation syndrome (OHSS). *Qatar Medical Journal.* 2002;11(2):28-31.

55. Fedder J, Loft A, Parner ET, Rasmussen S, Pinborg A. Neonatal outcome and congenital malformations in children born after ICSI with testicular or epididymal sperm: a controlled national cohort study. *Human reproduction (Oxford, England).* 2013;28(1):230-240.

56. Gambadauro P, Iliadis S, Brann E, Skalkidou A. Conception by means of in vitro fertilization is not associated with maternal depressive symptoms during pregnancy or postpartum. *Fertility and Sterility.* 2017;108(2):325-332.

57. Vakrilova L, Slavov S, Hitrova S, Slancheva B, Emilova Z. [Problems and neonatal outcome of very low birth weight newborn infants after in vitro fertilization]. *Akusherstvo i ginekologiia.* 2013;52(1):30-34.

58. Vincent-Rohfritsch A, Le Ray C, Anselem O, Cabrol D, Goffinet F. [Pregnancy in women aged 43 years or older: maternal and perinatal risks]. *Journal de gynecologie, obstetrique et biologie de la reproduction.* 2012;41(5):468-475.

59. Anzola AB, Pauly V, Geoffroy-Siraudin C, Gervoise-Boyer M-J, Montjean D, Boyer P. The first 50 live births after autologous oocyte vitrification in France. *Journal of assisted reproduction and genetics.* 2015;32(12):1781-1787.

60. Govaerts I, Devreker F, Koenig I, Place I, Van den Bergh M, Englert Y. Comparison of pregnancy outcome after intracytoplasmic sperm injection and in-vitro fertilization. *Human reproduction (Oxford, England).* 1998;13(6):1514-1518.

61. Tsutsumi R, Fujimoto A, Osuga Y, et al. Singleton pregnancy outcomes after assisted and non-assisted reproductive technology in infertile patients. *Reproductive Medicine and Biology.* 2012;11(3):149-153.

62. Vincent-Rohfritsch A, Le Ray C, Anselem O, Cabrol D, Goffinet F. Pregnancy in women aged 43 years or older: Maternal and perinatal risks. *Journal of Maternal-Fetal and Neonatal Medicine.* 2012;25(SUPPL. 2):99.

63. Malchau SS, Loft A, Larsen EC, et al. Perinatal outcomes in 375 children born after oocyte donation: A Danish national cohort study. *Fertility and Sterility.* 2013;99(6):1637.

64. Pochiraju M, Nirmalan PK. Type of conception and outcomes in women with singleton pregnancy. *Journal of Clinical and Diagnostic Research.* 2014;8(2):103-105.

65. Takahashi N, Miyake T, Nakamura H, et al. Obstetric and neonatal complications in pregnancies achieved by oocyte donation in our institution. *Journal of Obstetrics and Gynaecology Research.* 2018;44(8):1634.

66. Wang JX, Norman RJ, Kristiansson P. The effect of various infertility treatments on the risk of preterm birth. *Human Reproduction.* 2002;17(4):945-949.

67. Hasson J, Limoni D, Malcov M, et al. Obstetric and neonatal outcomes of pregnancies conceived after preimplantation genetic diagnosis: cohort study and meta-analysis. *Reproductive BioMedicine Online.* 2017;35(2):208-218.

68. Jing S, Li XF, Zhang S, Gong F, Lu G, Lin G. Increased pregnancy complications following frozen-thawed embryo transfer during an artificial cycle. *Journal of assisted reproduction and genetics.* 2019;36(5):925-933.

69. Loft A, Petersen K, Erb K, et al. A Danish national cohort of 730 infants born after intracytoplasmic sperm injection (ICSI) 1994-1997. *Human Reproduction.* 1999;14(8):2143-2148.

70. Yanaihara A, Yorimitsu T, Motoyama H, Ohara M, Kawamura T. Clinical outcome of frozen blastocyst transfer; single vs. double transfer. *Journal of assisted reproduction and genetics.* 2008;25(11-12):531-534.

71. Luke B, Gopal D, Cabral H, Stern JE, Diop H. Pregnancy, birth, and infant outcomes by maternal fertility status: the Massachusetts Outcomes Study of Assisted Reproductive Technology. *American journal of obstetrics and gynecology.* 2017;217(3):327.e321-327.e314.

72. Rakic S, Zecevic N, Jankovic-Raznatovic S, Vasiljevic M, Anicic R. Obstetric and neonatal outcomes in women aged 40 years or older after in vitro fertilization. *Clinical and experimental obstetrics & gynecology.* 2017;44(2):208-215.

73. Sabban H, Zakhari A, Patenaude V, Tulandi T, Abenhaim HA. Obstetrical and perinatal morbidity and mortality among in-vitro fertilization pregnancies: a population-based study. *Archives of gynecology and obstetrics.* 2017;296(1):107-113.

74. Sutcliffe AG, D'Souza SW, Cadman J, Richards B, McKinlay IA, Lieberman B. Outcome in children from cryopreserved embryos. *Archives of disease in childhood.* 1995;72(4):290-293.

75. Luke B, Stern JE, Kotelchuck M, et al. Birth outcomes by infertility treatment: Analyses of the massachusetts outcomes study of assisted reproductive technologies (MOSART). *Fertility and Sterility.* 2014;102(3 SUPPL. 1):e17.

76. Rakic S, Zecevic N, Jankovic-Raznatovic S, Vasiljevic M, Anicic R. Obstetric & neonatal outcomes in women aged 40 years or older after in vitro fertilization. *Clinical and Experimental Obstetrics and Gynecology.* 2017;44(2):208-215.

77. Sullivan EA, Chapman MG, Wang YA, Adamson GD. Population-based study of cesarean section after in vitro fertilization in Australia. *Birth (Berkeley, Calif).* 2010;37(3):184-191.

78. Bassiouny YA, Bayoumi YA, Gouda HM, Hassan AA. Is intracytoplasmic sperm injection (ICSI) associated with higher incidence of congenital anomalies? A single center prospective controlled study in Egypt. *The journal of maternal-fetal & neonatal medicine : the official journal of the European Association of Perinatal Medicine, the Federation of Asia and Oceania Perinatal Societies, the International Society of Perinatal Obstetricians.* 2014;27(3):279-282.

79. Agustsson T, Geirsson RT, Mires G. Obstetric outcome of natural and assisted conception twin pregnancies is similar. *Acta obstetricia et gynecologica Scandinavica.* 1997;76(1):45-49.

80. Reubinoff BE, Samueloff A, Ben-Haim M, Friedler S, Schenker JG, Lewin A. Is the obstetric outcome of in vitro fertilized singleton gestations different from natural ones? A controlled study. *Fertility and sterility.* 1997;67(6):1077-1083.

81. Sanchis Calvo A, Marcos Puig B, Juan Garcia L, et al. [Birth characteristics due to in vitro fertilization (IVF) techniques]. *Anales de pediatria (Barcelona, Spain : 2003).* 2009;70(4):333-339.

82. Xu XY, Yang JH, Ma XM, et al. Neonatal complication and birth defects in infants conceived by in vitro fertilization. *Chinese Journal of Contemporary Pediatrics.* 2015;17(4):350-355.

83. Xu X-Y, Yang J-H, Ma X-M, et al. [Neonatal complications and birth defects in infants conceived by in vitro fertilization]. *Zhongguo dang dai er ke za zhi = Chinese journal of contemporary pediatrics.* 2015;17(4):350-355.

84. Källén B, Finnström O, Nygren KG, Otterblad Olausson P, Wennerholm UB. In vitro fertilisation in Sweden: obstetric characteristics, maternal morbidity and mortality. *BJOG : an international journal of obstetrics and gynaecology.* 2005;112(1470-0328 (Print)).

85. Wennerholm UB, Hamberger L, Nilsson L, Wennergren M, Wikland M, Bergh C. Obstetric and perinatal outcome of children conceived from cryopreserved embryos. *Human Reproduction.* 1997;12(0268-1161 (Print)).

86. Wiklund I, Edman G, Larsson C, Andolf E. Personality and mode of delivery. *Acta obstetricia et gynecologica Scandinavica.* 2006;85(10):1225-1230.

87. Davies MJ, Moore VM, Willson KJ, et al. Reproductive Technologies and the Risk of Birth Defects. *Obstetrical & Gynecological Survey.* 2012;67(9):527-528.

88. Bay B, Ingerslev HJ, Lemmen JG, Degn B, Rasmussen IA, Kesmodel US. Preimplantation genetic diagnosis: a national multicenter obstetric and neonatal follow-up study. *Fertility and sterility.* 2016;106(6):1363-1369.e1361.
