# Supplementary material for: Caesarean section in pregnancies conceived by assisted reproductive technology: a systematic review and meta-analysis
Source: BMC Pregnancy Childbirth. 2021 Mar 22;21:244. doi: 10.1186/s12884-021-03711-x (PMC7986269; doi:10.1186/s12884-021-03711-x)
Supplement: Supplementary file 4 — Additional file 4. GRADE summary of quality of evidence. GRADE approach (Grading of Recommendations Assessment, Development, and Evaluation) [file 12884_2021_3711_MOESM4_ESM.docx]

| **GRADE approach** | | | | | |
| --- | --- | --- | --- | --- | --- |
| **Intervention**: ART  **Comparison**: spontaneous conception | | | | | |
| **Outcomes** | **Number of participants  (studies) Follow up** | **Certainty of the evidence (GRADE)** | **Relative effect (95% CI)** | **Anticipated absolute effects** | |
|  |  |  |  | **Risk with C-section in spontaneous conception** | **Risk difference with C-section total in IVF/ICSI** |
| C-Sections overall (IVF/ICSI vs Spontaneous conceptions) follow up: range 20 weeks to 39 weeks | 4010197 (34 observational studies) | ⨁⨁⨁◯ MODERATE ^a,b,c,d^ | **OR 1.90** (1.76 to 2.06) | 16 per 100 | **11 more per 100** (9 more to 12 more) |
| Elective C-Sections (IFV/ICSI vs Spontaneous Conceptions) follow up: range 20 weeks to 39 weeks | 364879 (10 observational studies) | ⨁⨁⨁◯ MODERATE ^d,e^ | **OR 1.91** (1.37 to 2.67) | 8 per 100 | **6 more per 100** (3 more to 11 more) |
| Emergent C-Section (IVF/ICSI vs Spontaneous conceptions) follow up: range 20 weeks to 39 weeks | 119248 (8 observational studies) | ⨁⨁⨁◯ MODERATE ^d,e^ | **OR 1.38** (1.09 to 1.75) | 14 per 100 | **4 more per 100** (1 more to 8 more) |
| C-section overall (IVF alone vs Spontaneous conceptions) follow up: range 20 weeks to 39 weeks | 3490789 (14 observational studies) | ⨁⨁⨁◯ MODERATE ^d,e,f^ | **OR 2.07** (1.86 to 2.30) | 15 per 100 | **12 more per 100** (10 more to 14 more) |
| C-section overall (ICSI vs Spontaneous conceptions) follow up: range 20 weeks to 39 weeks | 293113 (6 observational studies) | ⨁⨁⨁◯ MODERATE ^d,g,h^ | **OR 1.66** (1.29 to 2.15) | 26 per 100 | **11 more per 100** (5 more to 17 more) |
| C-section overall (Fresh embryo transfer after IVF/ICSI versus SC) follow up: range 20 weeks to 39 weeks | 2157788 (7 observational studies) | ⨁⨁◯◯ LOW ^d,i,j,k^ | **OR 1.55** (1.41 to 1.69) | 7 per 100 | **3 more per 100** (3 more to 4 more) |
| C-Sections overall (Frozen embryo transfer after IVF/ICSI versus SC) follow up: range 20 weeks to 39 weeks | 2086557 (6 observational studies) | ⨁⨁◯◯ LOW ^d,i,j,l^ | **OR 1.82** (1.65 to 2.01) | 16 per 100 | **10 more per 100** (8 more to 12 more) |
| ***The risk in the intervention group** (and its 95% confidence interval) is based on the assumed risk in the comparison group and the **relative effect** of the intervention (and its 95% CI).   **CI:** Confidence interval; **OR:** Odds ratio | | | | | |
| **GRADE Working Group grades of evidence** **High certainty:** We are very confident that the true effect lies close to that of the estimate of the effect **Moderate certainty:** We are moderately confident in the effect estimate: The true effect is likely to be close to the estimate of the effect, but there is a possibility that it is substantially different **Low certainty:** Our confidence in the effect estimate is limited: The true effect may be substantially different from the estimate of the effect **Very low certainty:** We have very little confidence in the effect estimate: The true effect is likely to be substantially different from the estimate of effect | | | | | |

#### Reason

a. Five studies were not adjusted for the outcome of interest (Beyer2016, Erntad 2016,katalinic 2004, Toshimitsu 2014, Olivennes 1993),

b. Two studies not reported the loss of follow-up (Liu 2015, Shevell 2005)

c. In three studies the selection of the control group was unclear (Apantaku 2008, Poikkeus 2007, liu 2015)

d. Heterogeneity I2 greater than 75%

e. Some studies showed non-significant effects when analyzed individually

f. Six studies were not matched

g. One study not reported the loss of follow-up and the selection of the control group was unclear (Farhi 2013)

h. One study did not present SC from the same community ( Farhi,2013), and another the ovulation induction or IUI was included in the control group (Pinborg 2010)

i. Missing data were imputed in some variables (Anzola2019)

j. Analysis of interest is unadjusted (Beyer, 2016)

k. Lost of follow up was not reported (Anzola, 2019)

l. The ovulation induction or IUI was included in the control group (Pinborg 2010)
